# Design, Synthesis, Antibacterial Evaluations and In Silico Studies of Novel Thiosemicarbazides and 1,3,4-Thiadiazoles

**DOI:** 10.3390/molecules27103161

**Published:** 2022-05-15

**Authors:** Sara Janowska, Dmytro Khylyuk, Sylwia Andrzejczuk, Monika Wujec

**Affiliations:** 1 Department of Organic Chemistry, Faculty of Pharmacy, Medical University, 20-093 Lublin, Poland; sara.janowska@umlub.pl (S.J.); dmytro.khylyuk@umlub.pl (D.K.); 2 Department of Pharmaceutical Microbiology, Faculty of Pharmacy, Medical University, 20-093 Lublin, Poland; sylwia.andrzejczuk@umlub.pl

**Keywords:** synthesis, thiosemicarbazide, 1,3,4-thiadiazole, antibacterial activity

## Abstract

The emergence of drug-resistant bacterial strains continues to be one of the major challenges of medicine. For this reason, the importance of searching for novel structures of antibacterial drugs chemically different from the currently known antibiotics is still of great importance. In this study, we synthesized the thiosemicarbazide and 1,3,4-thiadiazole derivatives and tested them for antibacterial activity. In in vitro tests, we examined the activity of the synthesized substances against Gram-positive and Gram-negative bacteria strains. While all 1,3,4-thiadiazoles tested lacked significant activity, the antimicrobial response of the thiosemicarbazides was moderate and it was also dependent on the type and position of the substituent on the phenyl ring. The highest activity towards all Gram-positive bacteria strains was shown by all three linear compounds containing the trifluoromethylphenyl group in the structure. The MIC (minimum inhibitory concentration) values were in the range of 3.9–250 µg/mL. Additionally, we try to explain the mechanism of the antibacterial activity of the tested compounds using the molecular docking to DNA gyrase and topoisomerase IV, following previous reports on the molecular basis of the activity of thiosemicarbazides. Docking simulations allow the purposing dual mechanism of the antibacterial activity of the synthesized compounds through inhibition of topoisomerase IV DNA gyrase with the moderate prevalence of the topoisomerase pathway.

## 1. Introduction

The introduction of penicillin to medical practice in 1942 started a scientific race in the development of new drugs effective in combating pathogenic bacteria. Along with the growing number of antibacterial drugs and the availability of antibiotics, the resistance of pathogens to therapeutic substances increased drastically [1]. Currently, despite a large number of known antibiotics and chemotherapeutic agents, the resistance of bacteria to antimicrobial agents has led to a situation in which it is justified to search for new classes of antibacterial drugs [1,2]. Antibiotic bacteria in hospitals are becoming an increasingly common phenomenon, and at the same time, drug-resistant strains of bacteria are gradually gaining resistance to all known antibacterial drugs. The main known method of fighting the problem of drug resistance of microorganisms is the search for new drugs with non-standard mechanisms of action [1].

**Figure 1:**
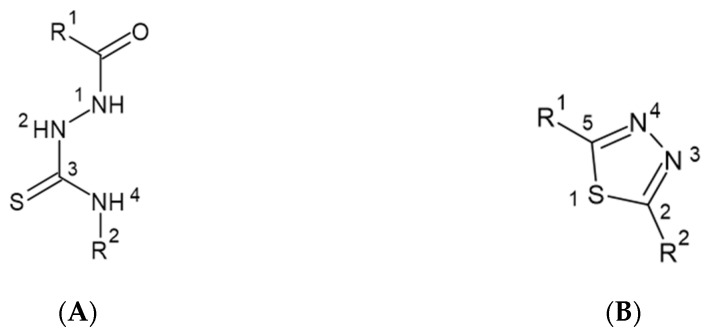
The general structure of thiosemicarbazide (**A**) and 1,3,4-thiadiazole (**B**) derivatives.

In recent years, scientists have been interested in the use of small organic molecules, including heterocyclic compounds, as promising therapeutic substances [3,4,5,6,7,8,9]. Literature data from our review indicate that 1,3,4-thiadiazole derivatives and intermediates in their synthesis, 1,4-disubstituted thiosemicarbazides, are commonly reported as promising antimicrobial agents [1,10,11,12,13,14,15,16,17,18,19]. Compounds containing these structures often show higher activity than standard antibacterial drugs, such as penicillin [10]. Thiosemicarbazide derivatives are commonly reported as anti-tuberculosis [20], antiviral [21], antimalarial and antibacterial [14,15,22] therapeutics. These compounds are synthesized not only as potential drugs, but also as precursors for the synthesis of heterocyclic compounds such as oxadiazoles, thiadiazoles and triazoles [22,23]. Thiosemicarbazides (Figure 1A) are the simplest hydrazine derivatives of thiocarbamic acid, containing NH_2_-NH-C (=S) NH_2_ moiety in the structure of molecules [24]. The 1,3,4-thiadiazoles (Figure 1B) formed as a result of their cyclization are molecules containing in their structure a five-membered heterocyclic ring composed of two nitrogen atoms and one sulfur atom [25].

The research work carried out by our team in previous years indicates the high potential of aryl thiosemicarbazides to inhibit type IIA topoisomerases (DNA gyrase and topoisomerase IV) [26,27,28,29]. This activity interferes with bacterial DNA synthesis, ultimately resulting in inhibition of their replication. Molecules having a thiosemicarbazide moiety have a completely different chemical structure than known antibacterial substances that target bacterial topoisomerase IV (e.g., quinolones). For this reason, this group of compounds has the potential to be used as new type of drugs in the fight against the problem of infectious diseases caused by drug-resistant bacteria [29]. Continuing our previous works, we decided to synthesize and test the antimicrobial activity of a series of new aryl thiosemicarbazide derivatives and their cyclic analogs—1,3,4-thiadiazoles. We planned the synthesis of a series of compounds having a 3-methoxyphenyl substituent on position 1 of thiosemicarbazide while on position 4 a phenyl ring with electron-donating and electron-withdrawing substituents. In this way, we wanted to find the relationship between structure and activity in the series of the tested compounds. A series of thiosemicarbazides were synthesized by the conventional method from the appropriate hydrazide and isothiocyanate. Then 1,3,4-thiadiazoles were obtained from thiosemicarbazides by cyclization with concentrated sulfuric acid. The structures of the compounds obtained were confirmed by infrared (IR) and nuclear magnetic resonance (NMR) spectra, and elemental analyses. The antibacterial activity of the synthesized compounds was assessed in vitro using the broth microdilution method against Gram-negative and Gram-positive reference strains bacterial species.

In our research, we also tried to answer questions about the mechanism of action of the newly synthesized compounds using the molecular docking technique. As the most probable strategy, based on previous works, we considered the inhibition of topoisomerases IIA.

## 2. Results and Discussion

### 2.1. Chemistry

**Scheme 1:**
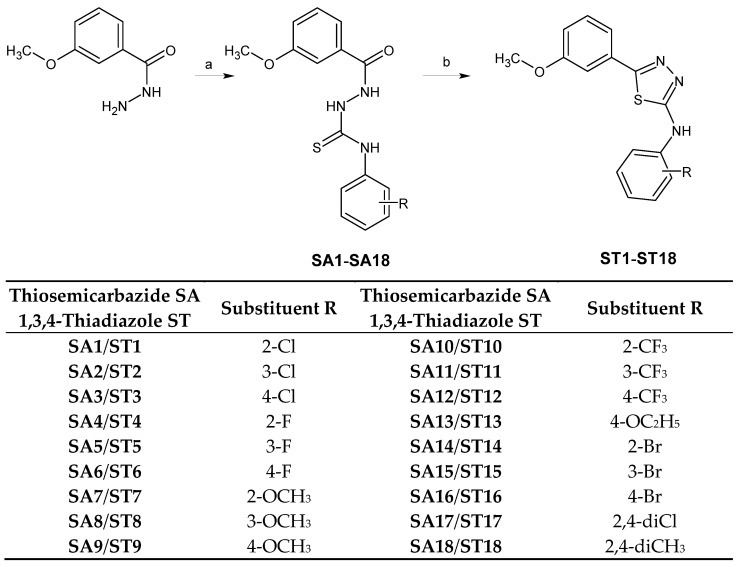
Synthesis of thiosemicarbazide **SA** and 1,3,4-thiadiazole **ST** derivatives. Reagents and conditions: (**a**) aryl isothiocyanate, 96% EtOH, reflux (**b**) H_2_SO_4 (conc)_, rt.

The synthesis of thiosemicarbazides and their cyclic analogs with the 1,3,4-thiadiazole ring was carried out according to the synthetic route presented in Scheme 1. The compounds tested include both newly synthesized molecules (**SA13**-**SA18**, **ST13**-**ST18**) and previously obtained derivatives (**SA1**-**SA12**, **ST1**-**ST12**). In the previously published work, we presented the antitumor activity of some of the 1,3,4-thiadiazoles [30] presented in this article. One of them, ST10 (5-(3-methoxyphenyl)-2-(2-trifluoromethylphenylamino)-1,3,4-thiadiazole) showed in the breast cancer cell lines (MCF-7 and MDA-MB-231) the strongest antiproliferative activity [30]. The evaluation of the antimicrobial activity of all synthesized molecules was described by us for the first time.

The reaction of 3-methoxyphenylhydrazide with the appropriate aryl isothiocyanate in refluxing ethanol gave 1-(3-methoxyphenyl)-4-aryl-thiosemicarbazides (**SA**) with good yields (43–95%). The obtained compounds were cyclized in concentrated sulfuric acid to the corresponding 1,3,4-thiadiazoles (**ST**), which were obtained with a yield of 22–70%. The structures of the new compounds described for the first time were determined using the IR, ^1^H NMR, ^13^C NMR spectroscopy and elemental analyzes.

^1^H NMR spectra showed chemical shifts of N1, N2 and N4 protons between 9.48 and 9.66; between 9.68 and 9.95; and between 10.49 and 10.62 ppm, as two or three singlets which confirmed the formation of the thiosemicarbazide scaffold (**SA13**-**SA18**). ^1^H NMR spectra confirmed the cyclization and the preparation of new 1,3,4-thiadiazole derivatives from the previously synthesized thiosemicarbazides. N1, N2 and N4 protons characteristic for thiosemicarbazides were not detected in 1,3,4-thiadiazole compounds. In the ^1^H NMR spectra of compounds **ST13**-**ST18**, the signals of the protons of the amine group between 10.34 and 10.79 ppm were visible. In two cases, we did not observe this proton. This is possible for labile protons that are bonded to the nitrogen atom. In ^1^H NMR spectra, the protons of the OCH_3_ group of all-new synthesized compounds show singlet signal at δ ~3.80–3.87 ppm. The ^13^C NMR spectra of the carbons of the compounds showed the carbon signals due to the resonance of the methoxy group in the range 55.75–55.84 ppm. The remaining carbon signals are appropriate for confirming the structure of the new compounds. In IR spectra of thiosemicarbazide derivatives absorption bands for C=O at 1664–1689 cm^−1^ were observed.

The ^1^H NMR and ^13^C NMR spectra for all new compounds are presented in Supplementary Materials.

### 2.2. Antibacterial Evaluation

The antibacterial activity of the synthesized thiosemicarbazides and 1,3,4-thiadiazole derivatives was tested based on the inhibition of bacterial growth in vitro using the broth microdilution method. The antibacterial results (MIC value) are reported in Table 1. Compounds with minimum inhibitory concentration (MIC) values of 1000 μg/mL and above are not included in the Table 1. (Table with all compounds is in Supplementary Materials).

All tested compounds showed a lack of antimicrobial activity against the reference strains of Gram-negative oxidase-negative *Klebsiella pneumoniae* ATCC 13883 and *Escherichia coli* ATCC 25922 bacteria, and oxidase-positive *Pseudomonas aeruginosa* ATCC 27853 bacteria. According to our screening results, some of the tested thiosemicarbazides showed potential antimicrobial activity against selected Gram-positive bacteria including the Gram-positive strains of *Staphylococcus aureus* ATCC 25923, *Staphylococcus aureus* ATCC 43300, *Staphylococcus epidermidis* ATCC 12228 and *Micrococcus luteus* ATCC 10240. The reference drugs used in our investigations were ciprofloxacin and cefuroxime. The MIC values for this antibiotics were 0.24–0.98 µg/mL and 0.24–62.5 µg/mL, respectively.

1,3,4-thiadiazole derivatives (**ST**) showed a lack or negligible antibacterial activity against both Gram-positive and Gram-negative strains, except for compounds with 4-bromophenyl substituent (**ST16)**, which showed promising activity against *S. epidermidis* ATCC 12228 with MIC value of 31.25 µg/mL, and *M. luteus* ATCC 10240 with MIC value of 15.63 µg/mL.

According to our results, the highest antibacterial activity against two bacterial *S. aureus* strains (*S. aureus* ATCC 25923 (MSSA, methicillin-susceptible) and *S. aureus* ATCC 43300 (MRSA, methicillin-resistant)) was demonstrated by thiosemicarbazide **SA1**, with a MIC value of 62.5 µg/mL against both strains. This compound has a chlorine atom in position 2 in the phenyl ring. The introduction of a second chlorine atom into the phenyl ring (**SA17**) results in a loss of activity against the MRSA strain. The thiosemicarbazides **SA10**, **SA11**, **SA14** with MIC values ranging from 125 µg/mL to 250 µg/mL also showed significant activity against both strains. Compounds with chlorine atom in position para in the phenyl ring (**SA3** and **ST3**) are two times more active towards MRSA than MSSA. Against *S. epidermidis* ATCC 12228 the highest activity with a MIC value of 31.25 µg/mL was demonstrated by **SA11**, **ST7**, **ST8**, and **ST16**. It is the only bacterial strain that is more sensitive to cyclic compounds than their linear precursors. Compounds with various substituents (electron-withdrawing—Cl, CF_3_, F and electron-donating—OCH_3_) in position ortho **SA1**, **SA7**, **SA10**, **ST4** showed antibacterial activity against *S. epidermidis* ATCC 12228 with a MIC value of 62.5 µg/mL. The strain most sensitive to the tested compounds turned out to be *M. luteus* ATCC 10240. The highest activity against these bacterial strains with a MIC value of 3.9 µg/mL was shown by the **SA11** and **SA12** thiosemicarbazides. The compound that showed the highest activity against all tested Gram-positive strains (MIC values ranging from 3.9 to 250 µg/mL) was **SA11**, thiosemicarbazide having a 3-trifluoromethylphenyl substituent. Unfortunately, despite testing a large number of compounds, it is not possible to derive a relationship between structure and activity in the studied groups of derivatives.

Minimal bactericidal concentration (MBC) was detected for compounds with MIC value ≤ 250 µg/mL. The MBC determined for the selected compounds (including of SA10, SA11 SA12, SA14, SA15, SA16, ST16, and ST7) against *M. luteus* ATCC 10240 resulted mainly equivalent concentration to MIC indicating that they are bactericidal rather than bacteriostatic agents. According to presented data, the MBC of tested compounds does not usually exceed by a factor greater than one to four times higher than their respective MIC values against only *M. luteus* ATCC 10240 bacteria, indicating their bactericidal effect. The MBC determined against staphylococci resulted from 16 to more times higher compared to MIC, indicating their bacteriostatic effect against these Gram-positive bacteria.

In conclusion, most of the tested thiosemicarbazides (**SA**) showed the ability to inhibit the growth of Gram-positive bacterial strains. The results are interesting for MRSA, the resistant subpopulation of a major pathogen that is associated with serious community-acquired and nosocomial diseases. This species can cause a large number of worldwide diseases and is important especially for hospital-associated infections [31].

In 2022, M. İhsan Han et al., published an article on the antibacterial activity of compounds from the group of thiosemicarbazides obtain from naproxen. The tests showed a moderate potential of the tested compounds against *S. aureus* ATCC 29213 and MRSA. The demonstrated MIC value against two strains of *S. aureus* was similar to that demonstrated by our compounds and was in the range of 64–256 μg/mL [19]. D. Bhakiaraj et al., in a study from 2021 also showed the potential of compounds from the group of thiosemicarbazides with tetrazole ring against strains belonging to *S. aureus* [17]. In 2022, Halit Muğlu et al., published a study on the antibacterial activity of compounds from the 1,3,4-thiadiazole group with thiophene moiety. The most active compound synthesized by them showed a MIC of 41 μg/mL against the strain *S. aureus* ATCC 25923 [16]. Unfortunately, none of the 1,3,4-thiadiazoles we synthesized showed similar activity.

Most of the tested 1,3,4-thiadiazole derivatives (**ST**) did not inhibit the growth of Gram-positive and Gram-negative bacteria. These compounds were previously synthesized by us and tested as potential anti-cancer drugs [30]. 1,3,4-thiadiazole derivatives, which in previous studies showed cytotoxic potential, showed no or negligible antibacterial activity in microbiological tests. In the case of substances that are potential cytostatics, the lack of activity against the bacterial microbiota may be an advantage and indicate the selectivity of action. 

### 2.3. Docking

Estimations of possible inhibition activity to the topoisomerase IV and DNA gyrase were made in comparison to the binging energies of synthesized compounds and reference literature reported inhibitors from the spectrums [32,33]. Calculated binding energies and inhibition constants are summarized in Table 2. Docking simulations allow the purposing dual mechanism of the antibacterial activity of the synthesized compounds through inhibition of Topoisomerase IV DNA gyrase with the moderate prevalence of the topoisomerase pathway.

**Figure 2:**
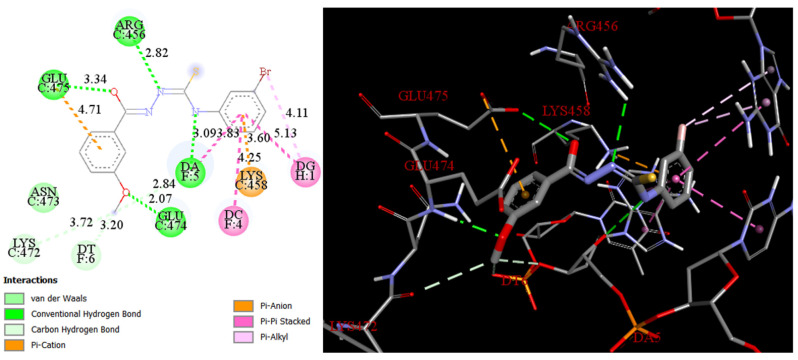
2D & 3D interaction maps of **SA15** with topoisomerase IV (PDB 3LTN).

Thiosemicarbazides demonstrate better binding energies compared to 1,3,4-thiadiazoles analogs. In silico calculations reveal higher binding energy for **SA15** than the potent topoisomerase IV inhibitor PD 0305970 [33]. Compound form 4 hydrogen bonds with Arg456 (2.82 Å), Glu475 (3.34 Å), Glu474 (2.07 Å), and DA (3.09 Å) (Figure 2). 3-Methoxyphenyl and 3-bromophenyl cores form different types of hydrophobic interactions with Glu475, Lys458, and nucleosides DC and DG respectively. **SA15** showed moderate antimicrobial activity, compared to the reference antimicrobial drugs ciprofloxacin and cefuroxime. The reason may be the absence of interaction with one of the key residues Arg117, which is necessary for the stabilization of the DNA cleavage complex.

One of the most active compounds **SA14** forms two hydrogen bonds between the sulfur atom and Gly459 (2.71 Å) and DG (3.28 Å). 3-Methoxyphenyl and 2-bromophenyl cores make the number of lipophilic interactions (Pi-alkyl, Pi-Pi Stacked, and Pi-Pi T-shaped) with the nucleosides. Arg122 binds to Br by a halogen bond with the length 2.61 Å. Summary interaction energy increases owing to the carbon-hydrogen bond and Pi-donor hydrogen bond.

As a result of in silico simulation **SA14** and **SA15** allow considered as interesting building blocks for further development of potent topoisomerase IV and DNA gyrase inhibitors.

## 3. Experimental

### 3.1. Chemistry

#### 3.1.1. General Comments

All of the substances were purchased from Sigma-Aldrich (Munich, Germany) and were used without further purification. The ^1^H NMR and ^13^C NMR spectra were recorded on the Bruker Avance 300 (Bruker BioSpin GmbH, Rheinstetten, Germany) in DMSO-d_6_. IR spectra were recorded on Spectrometer FT-IR Nicolett 8700 (Thermo Scientific, Waltham, MA, USA) The melting points were determined on the Stuart SMP50 melting point apparatus (Cole Parmer Ltd., Stone, UK) and are uncorrected. The purity of the compounds and the progress of the reaction were monitored by TLC (aluminum sheet 60 F254 plates (Merck Co., Kenilworth, NJ, USA). We used the solvent system CHCl_3_/EtOH (10:1, *v*/*v*). The elemental analyses were determined by a Perkin Elmer 2400 Series II CHNS/O analyzer (Waltham, MA, USA).

Compounds **SA1-SA12** and **ST1-ST12** were obtained and described in our previous work [30].

#### 3.1.2. Synthesis of Thiosemicarbazide Derivatives SA13-SA18

0.01 mol of 3-methoxybenzhydrazide and 5 mL of 96% ethanol were placed in a round bottom flask. It was heated under reflux until a clear solution was obtained. Next, an equimolar amount of the appropriate aryl isothiocyanate was added. The mixture was heated at the boiling point for 30 min (for compound **SA16**) or 1 h (for the rest derivatives). Compound **SA16** immediately precipitated. The solution was then cooled to room temperature until the product precipitated. The resulting solid was filtered off and washed with water and diethyl ether.

Detailed physicochemical properties of novel thiosemicarbazides:

**4-(4-Ethoxyphenyl)-1-(3-methoxyphenyl) thiosemicarbazide (SA13)**

Yield 71%, m.p.188–191 °C. IR (cm^−1^) KBr: 3151 (NH), 2919 (CH aliph.), 1666 (C=O), 1579 (CH arom.), 1336 (C=S), 1257 (C-O-C). ^1^H NMR (DMSO-d_6_)δ(ppm): 1.32 (t, 3H, CH_3_, *J* = 7.0 Hz), 3.82 (s, 3H, OCH_3_), 4.01 (q, 2H, CH_2_, *J* = 6.9 Hz), 6.88 (d, 2H, ArH, *J* = 9.0 Hz), 7.14–7.16 (m, 1H, ArH), 7.26 (bs, 2H, ArH), 7.41 (t, 1H, ArH, *J* = 7.9 Hz), 7.51–7.54 (m, 2H, ArH), 9.61 (s, 1H, NH), 9.68 (s, 1H, NH), 10.49 (s, 1H, NH). ^13^C NMR (DMSO-d_6_) δ (ppm): 15.1; 55.8; 63.5; 113.5; 114.1; 118.1; 120.6; 128.1; 129.9; 132.4; 134.4; 156.5; 159.5; 166.2. Elemental analysis for C_17_H_19_N_3_O_3_S. Calculated: C 59.11; H 5.54; N 12.17. Found: C 59.29; H 5.77; N 12.43.

**4-(2-Bromophenyl)-1-(3-methoxyphenyl) thiosemicarbazide (SA14)**

Yield 80%, m.p.142–145 °C. IR (cm^−1^) KBr: 3187 (NH), 2965 (CH aliph.), 1678 (C=O), 1582 (CH arom.), 1332 (C=S), 1285 (C-O-C). ^1^H NMR (DMSO-d_6_) δ(ppm): 3.82 (s, 3H, OCH_3_), 7.14–7.22 (m, 2H, ArH), 7.37–7.39 (m, 1H, ArH), 7.41–7.43 (m, 1H, ArH), 7.45–7.51 (m, 1H, ArH), 7.51–7.56 (m, 2H, ArH), 7.65 (d, 1H, ArH, *J* = 8.0 Hz), 9.64 (s, 1H, NH), 9.85 (s, 1H, NH), 10.61 (s, 1H, NH). ^13^C NMR (DMSO-d_6_) δ (ppm): 55.77; 113.7; 118.2; 120.7; 122.7; 128.1; 128.7; 130.0; 131.3; 132.9; 134.3; 153.2, 159.5. Elemental analysis for C_15_H_14_BrN_3_O_2_S. Calculated: C 47.37; H 3.71; N 11.05. Found: C 47.60; H 3.83; N 11.35.

**4-(3-Bromophenyl)-1-(3-methoxyphenyl) thiosemicarbazide (SA15)**

Yield 79%, m.p.165–167 °C. IR (cm^−1^) KBr: 3150 (NH), 2959 (CH aliph.), 1667 (C=O), 1583 (CH arom.), 1351 (C=S), 1295 (C-O-C). ^1^H NMR (DMSO-d_6_) δ(ppm): 3.82 (s, 3H, OCH_3_), 7.16–7.17 (m, 1H, ArH), 7.30 (t, 1H, *J* = 8.2 Hz) 7.34–7.36 (s, 1H, ArH), 7.43 (t, 1H, ArH, *J* = 7.9 Hz), 7.51–7.55 (s, 3H, ArH), 7.72 (s, 1H, ArH), 9.90 (s, 2H, 2NH), 10.56 (s, 1H, NH). ^13^C NMR (DMSO-d_6_) δ (ppm): 55.8; 113.6; 118.2; 120.6; 125.2; 128.3 (d, *J* = 70.8 Hz), 130.1 (d, *J* = 51.5 Hz); 134.2; 141.4; 159.6; 166.2. Elemental analysis for C_15_H_14_BrN_3_O_2_S. Calculated: C 47.37; H 3.71; N 11.05. Found: C 47.50; H 3.65; N 11.40.

**4-(4-Bromophenyl)-1-(3-methoxyphenyl) thiosemicarbazide (SA16)**

Yield 66%, m.p.173–175 °C.IR (cm^−1^) KBr: 3150 (NH), 2969 (CH aliph.), 1664 (C=O), 1585 (CH arom.), 1346 (C=S), 1259 (C-O-C). ^1^H NMR δ(DMSO-d_6_) δ(ppm): 3.81 (s, 3H, OCH_3_), 7.13–7.17 (m, 1H, ArH), 7.39–7.44 (m, 3H, ArH), 7.50–7.54 (m, 4H, ArH), 9.88 (s, 2H, 2NH), 10.59 (s, 1H, NH). ^13^C NMR (DMSO-d_6_) δ (ppm): 55.8; 113.5; 118.2; 120.6; 127.1; 129.9; 131.3; 133.7; 139.1; 159.5; 166.1. Elemental analysis for C_15_H_14_BrN_3_O_2_S. Calculated: C 47.37; H 3.71; N 11.05. Found: C 47.52; H 3.85; N 11.35.

**4-(2,4-Dichlorophenyl)-1-(3-methoxyphenyl) thiosemicarbazide (SA17)**

Yield 55%, m.p.172–175 °C. IR (cm^−1^) KBr: 3196 (NH), 2936 (CH aliph.), 1668 (C=O), 1582 (CH arom.), 1345 (C=S), 1259 (C-O-C). ^1^H NMR (DMSO-d_6_) δ (ppm): 3.87 (s, 3H, OCH_3_), 7.13–7.16 (m, 1H, ArH), 7.39–7.44 (m, 3H, ArH), 7.52–7.55 (m, 2H, ArH), 7.66 (s, 1H, ArH), 9.66 (s, 1H, NH), 9.95 (s, 1H, NH), 10.62 (s, 1H, NH). ^13^C NMR (DMSO-d_6_) δ (ppm): 55.8; 113.7; 118.2; 120.7; 127.7; 129.2; 129.9; 131.9; 132.7; 133.0; 134.2; 136.7; 159.5; 166.4; 182.5. Elemental analysis for C_15_H_13_Cl_2_N_3_O_2_S. Calculated: C 48.66; H 3.54; N 11.35. Found: C 48.86; H 3.75; N 11.70.

**1-(3-Methoxyphenyl)-4-(2,4-dimethylphenyl) thiosemicarbazide (SA18)**

Yield 62%, m.p.165–167 °C. IR (cm^−1^) KBr: 3164 (NH), 2961 (CH aliph.), 1689 (C=O), 1583 (CH arom.), 1329 (C=S), 1267 (C-O-C). ^1^H NMR (DMSO-d_6_) δ (ppm): 2.13 (s, 3H, CH_3_), 2.26 (s, 3H, CH_3_), 3.80 (s, 3H, OCH_3_), 6.97–7.02 (m, 2H, ArH), 7.11–7.14 (m, 1H, ArH), 7.35-7.42 (m, 2H, ArH), 7.50–7.53 (m, 2H, ArH), 9.48 (s, 1H, NH), 9.76 (s, 1H, NH), 10.49 (s, 1H, NH). ^13^C NMR (DMSO-d_6_) δ (ppm): 18.1; 21.1; 55.8; 113.7; 118.0; 120.7; 126.8; 129.1; 131.0; 134.4; 136.0; 136.1; 159.5; 166.3; 182.3. Elemental analysis for C_17_H_19_N_3_O_4_S. Calculated: C 56.50; H 5.30; N 11.63. Found: C 56.83; H 5.43; N 11.70.

#### 3.1.3. Synthesis of 5-(3-Methoxyphenyl)-2-Substituted-1,3,4-Thiadiazole ST13-ST18

To 0.2 g of the thiosemicarbazide derivatives, 0.5 mL of concentrated sulfuric acid (VI) was added dropwise. The reaction mixture was stirred until the precipitate dissolved. Next, the solution was left for 2 h at room temperature. Then, crushed ice was added to the reaction flasks and mixed intensively. The precipitate formed which was allowed to dissolve the ice at room temperature. The solid product was filtered off, dried thoroughly with filter paper, and crystallization from butanol.

Detailed physicochemical properties of novel 1,3,4-thiadiazoles:

**2-(4-Ethoxyphenylamino)-5-(3-methoxyphenyl)-1,3,4-thiadiazole (ST13)**

Yield 37% m.p.171–173 °C. IR (cm^−1^) KBr: 3191 (NH), 2838 (CH aliph.), 1608 (C=N), 1592 (CH arom.), 1278 (C-O-C), 761 (C-S). ^1^H NMR (DMSO-d_6_) δ (ppm):1.32 (t, 3H, CH_3_, *J* = 7.0 Hz), 3.83 (s, 3H, OCH_3_), 4.00 (q, 2H, CH_2_, *J* = 7.0 Hz), 6.93 (d, 2H, ArH, *J* = 9.0 Hz), 7.03–7.07 (m, 1H, ArH), 7.35–7.39 (m, 2H, ArH), 7.41–7.44 (m, 1H, ArH), 7.53 (d, 2H, ArH, *J* = 9.0 Hz), 10.34 (s, 1H, NH). ^13^C NMR (DMSO-d_6_) δ (ppm): 15.2; 55.8; 63.7; 111.7; 115.4; 116.4; 119.9; 130.9; 132.2; 134.4; 154.5; 157.0; 160.1; 165.3. Elemental analysis for C_17_H_17_N_3_O_2_S. Calculated: C 62.36; H 5.23; N 12.83. Found: C 62.48; H 5.34; N 12.85.

**2-(2-Bromophenylamino)-5-(3-methoxyphenyl)-1,3,4-thiadiazole (ST14)**

Yield 14%, m.p.102–105 °C. IR (cm^−1^) KBr: 3163 (NH), 2833 (CH aliph.), 1648 (C=N), 1578 (CH arom.), 1262 (C-O-C), 757 (C-S). ^1^H NMR (DMSO-d_6_) δ(ppm): 3.83 (s, 3H, OCH_3_), 7.06–7.09 (m, 2H, ArH), 7.11 (d, 1H, *J* = 7.5 Hz), 7.37–7.39 (m, 2H, ArH), 7.40–7.45 (m, 2H, ArH), 7.70 (d, 1H, *J* = 8.0 Hz), 10.60 (s, 1H, NH). ^13^C NMR (DMSO-d_6_) δ (ppm): 55.8; 111.8, 114.8 (d, *J* = 27.4 Hz); 115.6; 116.5 (d, *J* = 23.4 Hz); 119.8; 123.4, 124.0; 125.5; 126.2; 129.0 (d, *J* = 17.9 Hz); 129.8; 130.9; 132.0; 133.6 (d, *J* = 15.5 Hz); 139.3; 159.5; 160.1; 168.3. Elemental analysis for C_15_H_12_BrN_3_OS. Calculated: C 49.73; H 3.34; N 11.60. Found: C 50.01; H 3.54; N 11.86.

**2-(3-Bromophenylamino)-5-(3-methoxyphenyl)-1,3,4-thiadiazole (ST15)**

Yield 28%, m.p.188–190 °C. IR (cm^−1^) KBr: 3190 (NH), 2833 (CH aliph.), 1625 (C=N), 1581 (CH arom.), 1266 (C-O-C), 749 (C-S). ^1^H NMR (DMSO-d_6_) δ(ppm): 3.84 (s, 3H, OCH_3_), 7.07–7.11 (m, 1H, ArH), 7.20 (d, 1H, ArH, *J* = 9.7 Hz), 7.32 (t, 1H, ArH, *J* = 8.0 Hz), 7.42–7.44 (m, 3H, ArH), 7.48–7.51 (m, 1H, ArH), 8.10 (s, 1H, ArH), 10.79 (s, 1H, NH). ^13^C NMR (DMSO-d_6_) δ (ppm): 55.8, 111.9; 116.8 (d, *J* = 10.7 Hz); 120.1 (d, *J* = 32.7 Hz); 122.5; 124.9; 130.9; 131.5; 131.8; 142.3; 158.6; 160.1; 164.1. Elemental analysis for C_15_H_12_BrN_3_OS. Calculated: C 49.73; H 3.34; N 11.60. Found: C49.85; H 3.45; N 11.80. 

**2-(4-Bromophenylamino)-5-(3-methoxyphenyl)-1,3,4-thiadiazole (ST16)**

Yield 17%, m.p.218–220 °C. IR (cm^−1^) KBr: 3150 (NH), 2832 (CH aliph.), 1664 (C=N), 1598 (CH arom.), 1261 (C-O-C), 751 (C-S). ^1^H NMR (DMSO-d_6_) δ (ppm): 3.84 (s, 3H, OCH_3_), 7.08–7.10 (m, 1H, ArH), 7.41–7.45 (m, 3H, ArH), 7.54–7.55 (m, 2H, ArH), 7.64–7.66 (m, 2H, ArH), 10.70 (s, 1H, NH). ^13^C NMR (DMSO-d_6_) δ (ppm): 55.8; 113.6; 118.2; 120.6; 128.4; 129.9; 131.2; 134.2; 139.2; 159.5; 166.2. Elemental analysis for C_15_H_12_BrN_3_OS. Calculated: C 49.73; H 3.34; N 11.60. Found: C 49.94; H 3.38; N 11.68.

**2-(2,4-Dichlorophenylamino)-5-(3-methoxyphenyl)-1,3,4-thiadiazole (ST17)**

Yield 23%, m.p.163–165 °C. IR (cm^−1^) KBr: 3130 (NH), 2838 (CH aliph.), 1608 (C=N), 1563 (CH arom.), 1285 (C-O-C), 779 (C-S). ^1^H NMR (DMSO-d_6_) δ (ppm): 3.84 (s, 3H, OCH_3_), 7.08–7.10 (m, 1H, ArH), 7.28–7.30 (m, 1H, ArH), 7.39–7.40 (m, 1H, ArH), 7.45–7.47 (m, 2H, ArH), 7.47–7.49 (m, 1H, ArH), 8.36–8.39 (m, 1H, ArH). ^13^C NMR (DMSO-d_6_) δ (ppm): 55.8; 111.9; 116.6; 119.9; 123.0; 123.9; 127.3; 128.4; 129.5; 130.9; 131.8; 160.1; 164.7. Elemental analysis for C_15_H_11_Cl_2_N_3_OS. Calculated: C 51.15; H 3.15; N 11.93. Found: C 51.36; H 3.34; N 11.98.

**5-(3-Methoxyphenyl)-2-(2,4-dimetylphenylamino)-1,3,4-thiadiazole (ST18)**

Yield 34%, m.p.174–176 °C. IR (cm^−1^) KBr: 3153 (NH), 2829 (CH aliph.), 1634 (C=N), 1583 (CH arom.), 1259 (C-O-C), 766 (C-S). ^1^H NMR (DMSO-d_6_) δ (ppm): 2.25 (s, 3H, CH_3_), 2.27 (s, 3H, CH_3_), 3.82 (s, 3H, OCH_3_), 7.03 (m, 1H, ArH), 7.04–7.06 (m, 1H, ArH), 7.08–7.09 (m, 1H, ArH), 7.33–7.34 (m, 2H, ArH), 7.40 (t, 1H, ArH, *J* = 8.1 Hz), 7.60 (d, 1H, ArH, *J* = 8.1 Hz). ^13^C NMR (DMSO-d_6_) δ (ppm): 18.2; 20.9; 55.7; 111.6; 116.4; 119.7; 123.1; 127.7; 130.9; 131.9; 132.2; 134.5; 137.2; 157.1; 160.1; 167.9. Elemental analysis for C_17_H_17_N_3_O_3_S. Calculated: C 59.46; H 4.99; N 12.24. Found: C 59.56; H 5.21; N 12.45.

### 3.2. Microbiology

The antimicrobial activity of the thiosemicarbazides and 1,3,4-thiadiazoles series was tested against four Gram-positive reference strains (*S. aureus* ATCC 25923, *S. aureus* ATCC 43300, *S. epidermidis* ATCC 12228, *M.* *luteus* ATCC 10240) and three Gram-negative reference bacteria (*K. pneumoniae* ATCC 13883, *E. coli* ATCC 25922, *P. aeruginosa* ATCC 27853). These bacterial strains cause the most common infections among humans. To perform the assay, all microorganisms were placed in sterile physiological saline (0.85% NaCl) to obtain a suspension of the optical density of the 0.5 McFarland standard; 1.50 × 10 ^8^ colony-forming units (CFUs)/mL. A solution of the test compounds was prepared in Dimethyl sulfoxide (DMSO) at a concentration of 50 mg/mL. The solutions were then diluted with Mueller-Hinton broth to obtain a solution with a concentration of 2000 µg/mL. Then, using the two-fold dilution method in broth, the test compounds were introduced into the wells of the microplate to give a final concentration of 0.49 to 1000 µg/mL. Negative control without tested compounds was used. Cefuroxime and ciprofloxacin in a concentration 0.007–15.625 µg/mL were used as a positive controls. These antibiotics are most commonly used in the outpatient treatment of bacterial infections.

The in vitro antimicrobial activity of the tested compounds was determined by the MIC, which was defined as the lowest concentration of the compound at which no apparent growth of the test organisms was observed. MIC values were determined using the broth microdilution method following the CLSI recommendation [34]. MIC assays were in triplicate. MBC is defined as the lowest concentration of antimicrobial agent required to reduces the viability of the initial bacterial inoculum by ≥99.9%. Antimicrobial agents are usually regarded as bactericidal if the ratio of the MBC to MIC is lower than 4. For the MBC test, the agar plate assay was used. MBC was determined after the broth dilution period of a MIC test has been completed. After the incubation and MIC detection, 5 µL of the sample from each well of microplate were inoculated onto the agar plate containing the Mueller-Hinton Agar medium. The agar plate was then incubated at 35 °C for 24 h, which allowed the tested bacteria to grow on the agar media. The MBC values were determined by observing the first clear area on the agar plate that has no observable bacterial growth.

### 3.3. Docking

**Figure 3:**
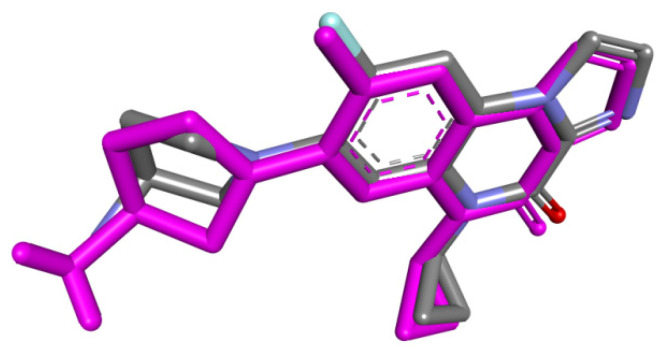
Overlying of real (grey-colored) and predicted position (pink-colored) of the ligand E32 (RMSD = 1.09) DNA gyrase (PDB code: 6FQM).

There are literature data, which describe 1,3,4-thiadiazole and thiosemicarbazide derivatives as potent DNA gyrase and topoisomerase IV inhibitors [26,33,35]. That’s why DNA gyrase (PDB code: 6FQM) [33] and topoisomerase IV (PDB code: 3LTN) [33] were prior enzymes for in silico docking simulations. Crystal structures were downloaded from the Protein Data Bank. Preparation of the selected enzymes includes deleting the water molecules and all co-crystallized entities from the protein’s structures. In addition, polar hydrogen atoms were added to the enzyme’s structures, nonpolar hydrogen atoms were merged, and Kollman charges were added and spread over the residues. All of the preparating procedures have been utilized with the using AutoDock Tools v.4.2.6 [36]. The chemical structures of the **SA**s and **ST**s were drawn by Biovia Draw [37] and Hyperchem software version 7.5 was used to optimize all of the compounds so as achieve steady conformations at a minimum energy level [38]. AM1 semi-empirical quantum technique was used for this energy minimizations until RMS gradients were obtained less than 0.01 kcal/(Å mol) and the electrostatic charge was assigned by the Gasteiger method [39]. The grid for the docking process was calculated using AutoGrid and its size was set to 50 × 50 × 50 points for DNA gyrase and 60 × 60 × 60 for topoisomerase IV in x, y and z directions. The Lamarckian genetic algorithm was selected for ligand conformational searching and docking parameters including 30 runs, 300 conformational possibilities, 50 populations, and 2,500,000 energy evaluations, a maximum number of 106 energy evaluations, a mutation rate of 0.02, and a crossover rate of 0.80 [40]. The method used for docking validation was overlaying real and predicted poses of the initial ligands into active sites of the DNA gyrase and topoisomerase IV proteins. Docking programs are preferred to predict results from experimental poses with RMSD no more than 2 Å [41]. Smaller RMSD indicates that the ligand position in redocking results is closer to crystallography results (Figure 3).

## 4. Conclusions

Continuing our investigation on the search for compounds with significant antibacterial activity, we obtained a number of new thiosemicarbazide and 1,3,4-thiadiazole derivatives with a 3-methoxyphenyl substituent. We tested them against selective Gram-positive and Gram-negative bacteria strains. Additionally, we included the previously obtained derivatives from these groups in the biological investigation. All tested compounds showed no activity against Gram-negative bacteria. Most of the tested thiosemicarbazides showed moderate activity against all strains of Gram-positive bacteria. The highest antibacterial activity against two bacterial *S. aureus* strains—*S. aureus* ATCC 25923 and *S. aureus* ATCC 43300 was demonstrated by 4-(2-chlorophenyl)-1-(3-methoxyphenyl) thiosemicarbazide **SA1**, with a MIC value of 62.5 µg/mL against both strains. The compound that showed the highest activity against all tested Gram-positive bacteria strains (MIC values ranging 3.9–250 µg/mL) was **SA11**, thiosemicarbazide having a 3-trifluoromethylphenyl substituent. A molecular docking study showed that the probable mechanism of action of the tested compounds is the inhibition of DNA gyrase and topoisomerase IV. Basically, the results obtained by us indicate a weak antimicrobial activity of the studied groups of compounds. It may be related both to the structure of the compounds and the bacterial strains used. In the future, it is possible to extend the research to other bacteria and introduce other pharmacophore groups (e.g., amine) to the phenyl ring.

## Figures and Tables

**Table 1:** Antibacterial data (MIC and MBC, μg/mL) for thiosemicarbazide **SA1**-**SA18** and 1,3,4-thiadiazole **ST1**-**ST18** derivatives and reference drugs.

Compounds	*Staphylococcus aureus* ATCC 25923		*Staphylococcus aureus* ATCC 43300		*Staphylococcus epidermidis* ATCC 12228		*Micrococcus luteus* ATCC 10240	
	MIC	MBC	MIC	MBC	MIC	MBC	MIC	MBC
**SA1**	62.5	>1000	62.5	>1000	62.5	>1000	62.5	>1000
**SA2**	1000	nd	>1000	nd	500	nd	15.63	>1000
**SA3**	1000	nd	500	nd	125	>1000	250	>1000
**SA4**	1000	nd	>1000	nd	1000	nd	>1000	nd
**SA5**	125	>1000	1000	nd	>1000	nd	>1000	nd
**SA6**	500	nd	1000	nd	250	>1000	250	>1000
**SA7**	250	>1000	1000	nd	62.5	1000	500	nd
**SA10**	250	>1000	250	>1000	62.5	>1000	15.63	15.63
**SA11**	250	>1000	250	>1000	31.25	>1000	3.9	3.9
**SA12**	500	nd	>1000	nd	125	>1000	3.9	3.9
**SA13**	1000	nd	500	nd	125	>1000	250	1000
**SA14**	125	>1000	250	>1000	125	>1000	250	250
**SA15**	500	nd	1000	nd	250	>1000	15.63	15.63
**SA16**	125	>1000	>1000	nd	500	nd	15.63	15.63
**SA17**	62.5	>1000	>1000	nd	>1000	nd	15.63	500
**ST2**	1000	nd	1000	nd	62.5	1000	500	nd
**ST3**	1000	nd	500	nd	500	nd	250	>1000
**ST4**	500	nd	>1000	nd	62.5	>1000	31.25	125
**ST6**	>1000	nd	500	nd	1000	nd	500	nd
**ST7**	>1000	nd	>1000	nd	31.25	>1000	31.25	31.25
**ST8**	>1000	nd	1000	nd	31.25	1000	500	nd
**ST12**	>1000	nd	1000	nd	250	>1000	1000	nd
**ST14**	>1000	nd	1000	nd	500	nd	>1000	nd
**ST15**	>1000	nd	500	nd	250	>1000	>1000	nd
**ST16**	250	>1000	500	nd	31.25	1000	15.63	15.63
**ST17**	250	>1000	1000	nd	500	>1000	125	125

nd, not determined. See Supplementary Materials.

**Table 2:** Calculated binding energies and inhibition constants for synthesized compounds.

Compounds	Topoisomerase IV (3LTN)		DNA Gyrase (6FQM)	
	Binding Energy Kcal/mol	Inhibition Constant, Ki µM	Binding Energy Kcal/mol	Inhibition Constant, Ki µM
**SA1**	−8.38	0.72	−7.76	2.06
**SA2**	−8.22	0.94	−8.40	0.7
**SA3**	−8.27	0.86	−7.97	1.44
**SA4**	−8.34	0.77	−7.66	2.42
**SA5**	−7.81	1.89	−7.92	1.57
**SA6**	−7.59	2.73	−7.64	2.53
**SA7**	−7.75	2.09	−6.90	8.79
**SA10**	−7.90	1.63	−7.53	3.05
**SA11**	−8.18	1.01	−6.93	8.36
**SA12**	−8.28	0.85	−8.06	1.23
**SA 13**	−8.24	0.91	−7.62	2.61
**SA14**	−7.86	1.74	−8.93	0.28
**SA15**	−9.11	0.21	−8.84	0.33
**SA16**	−7.72	2.21	−7.85	1.77
**SA17**	−8.74	0.39	−8.00	1.36
**ST2**	−8.58	0.51	−8.67	0.44
**ST3**	−8.58	0.51	−8.67	0.44
**ST4**	−8.12	1.12	−8.13	1.11
**ST6**	−7.44	3.52	−7.68	2.33
**ST7**	−8.22	0.93	−8.16	1.04
**ST8**	−8.56	0.53	−8.12	1.13
**ST12**	−7.25	4.88	−7.60	2.71
**ST14**	−8.31	0.81	−8.96	0.27
**ST15**	−8.64	0.46	−8.16	1.04
**ST16**	−7.72	2.21	−7.85	1.77
**ST17**	−7.85	1.76	−7.67	2.41
PD 0305970	−8.38	0.72	-	-
E32	-	-	−9.29	0.155

## Data Availability

The details of the data supporting the report results in this research were included in the paper and Supplementary Materials.

## Supplementary Materials

The following supporting information can be downloaded at: https://www.mdpi.com/article/10.3390/molecules27103161/s1, ^1^H NMR, ^13^C NMR spectra and full Table 1.
