# Supplementary material for: Design, Synthesis, Antibacterial Evaluations and In Silico Studies of Novel Thiosemicarbazides and 1,3,4-Thiadiazoles
Source: Molecules. 2022 May 15;27(10):3161. doi: 10.3390/molecules27103161 (PMC9147709; doi:10.3390/molecules27103161)
Supplement: Supplementary file 1 [file molecules-27-03161-s001.zip › molecules-1713138-supplementary.pdf]

Supplementary Information

Synthesis and antibacterial activity of thiosemicarbazide and 1,3,4-thiadiazole with 3-methoxyphenyl substituent.

Sara Janowska[1], Dmytro Khylyuk[1], Sylwia Andrzejczuk[2] and Monika Wujec[1,*]

[1]Department of Organic Chemistry, Faculty of Pharmacy, Medical University,
4a Chodzki Str., 20-093 Lublin, Poland;
sara.janowska@umlub.pl; dmytro.khylyuk@umlub.pl; monika.wujec@umlub.pl
[2]Department of Pharmaceutical Microbiology, Faculty of Pharmacy,
Medical University, 1 Chodzki Str., 20-093 Lublin, Poland;
sylwia.andrzejczuk@umlub.pl
*correspondence: monika.wujec@umlub.pl

Table of contents:

1. ^{1}H NMR spectra of thiosemicarbazide derivatives (SA13-SA18)……………………………………...……………………………………2
2. ^{1}H NMR spectra of 1,3,4-thiadiazole derivatives (ST13-ST18).……………………………………………………………………..8
3. ^{13}C NMR spectra of thiosemicarbazide derivatives (SA13-SA18)……….………………………………………………………....14
4. ^{13}C NMR spectra of 1,3,4-thiadiazole derivatives (ST13-ST18)……………..…………………………………………………........20
5. Tabele antibacterial activity of compounds SA1-18 and ST1-18 against Gram-positive strains………………………………………..26
6. Tabele antibacterial activity of compounds SA1-18 and ST1-18 against Gram-negative strains………………………………………28

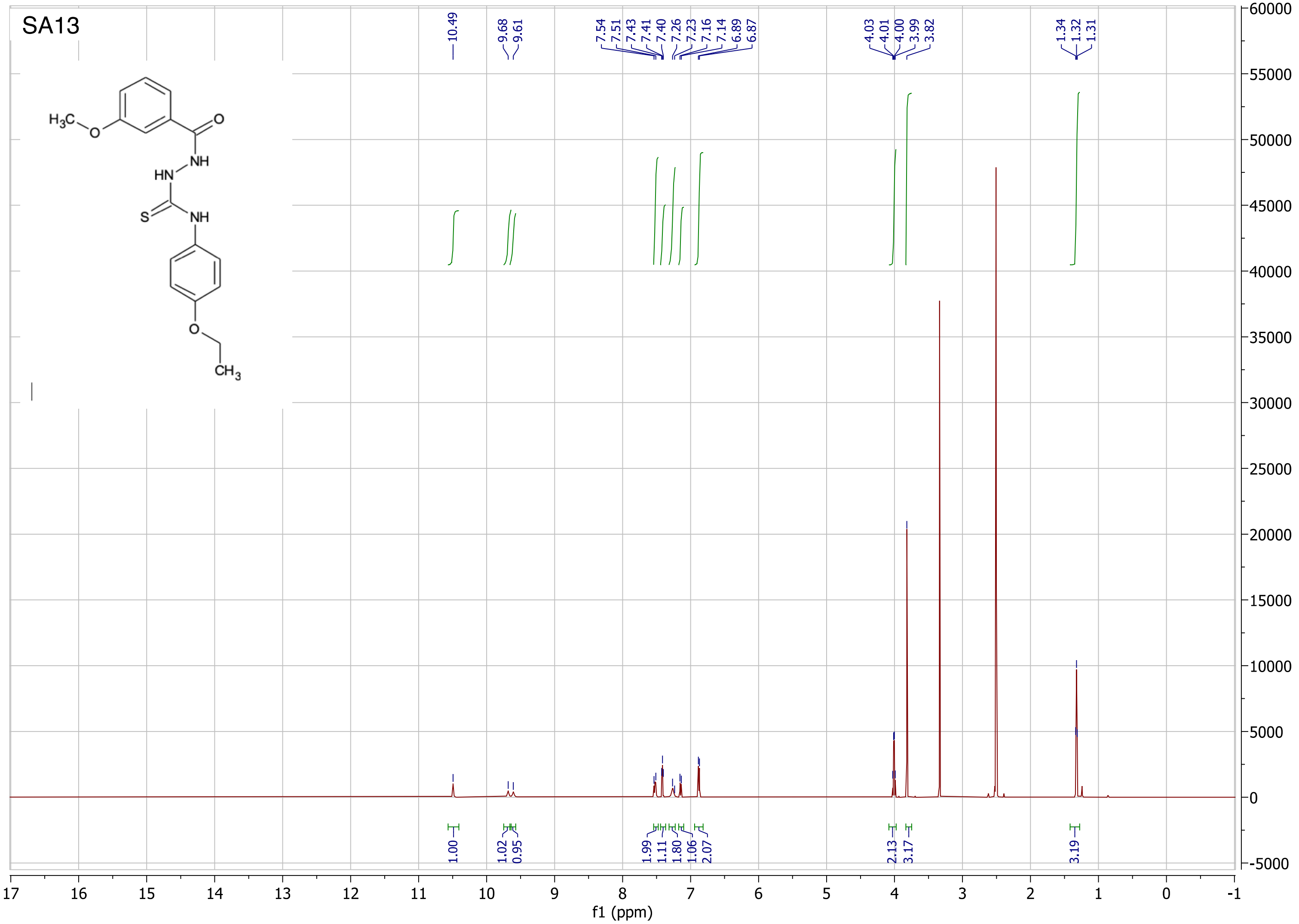

Figure S1. The ^{1}H NMR of compound SA13.

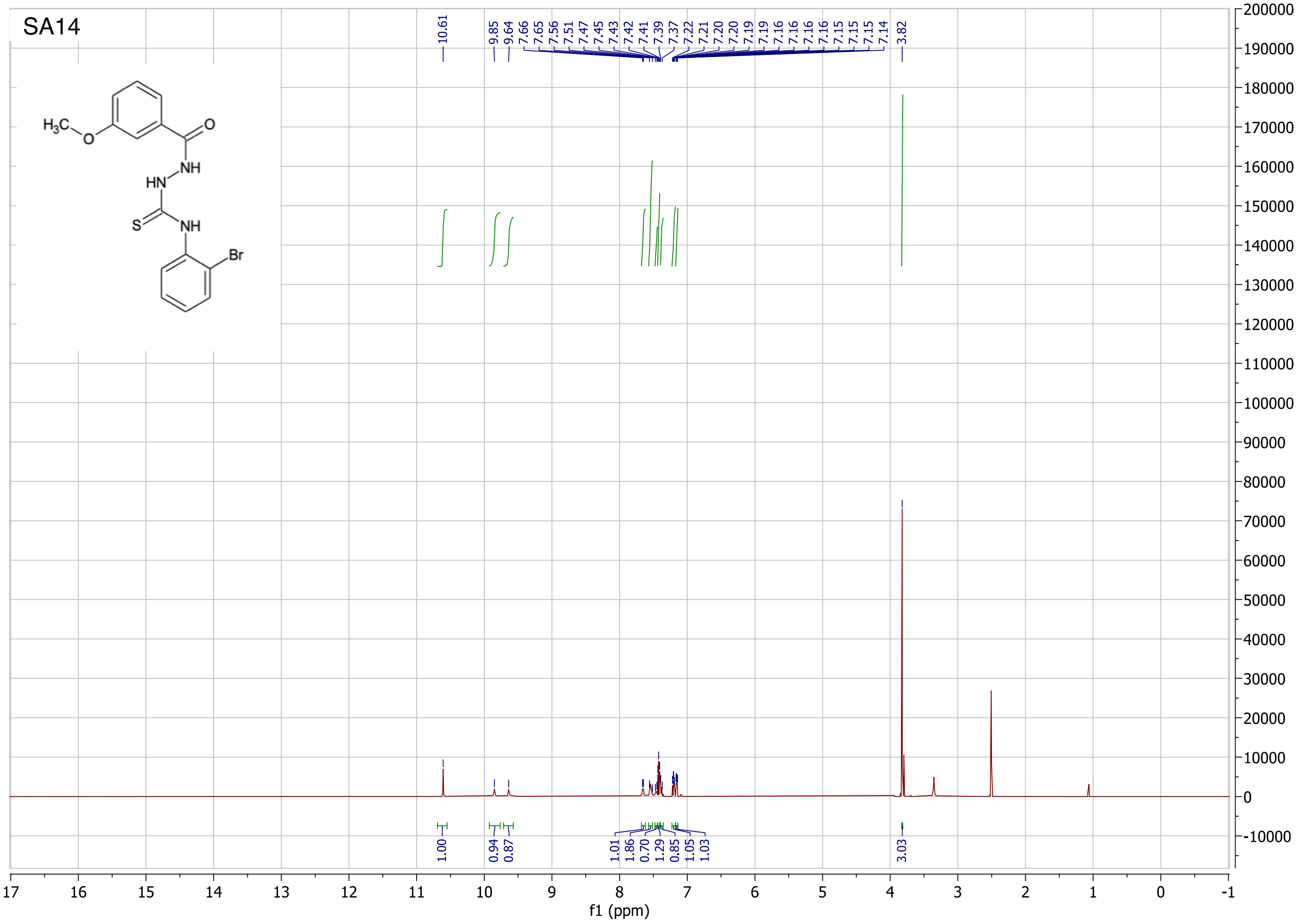

Figure S2. The ¹H NMR of compound SA14.

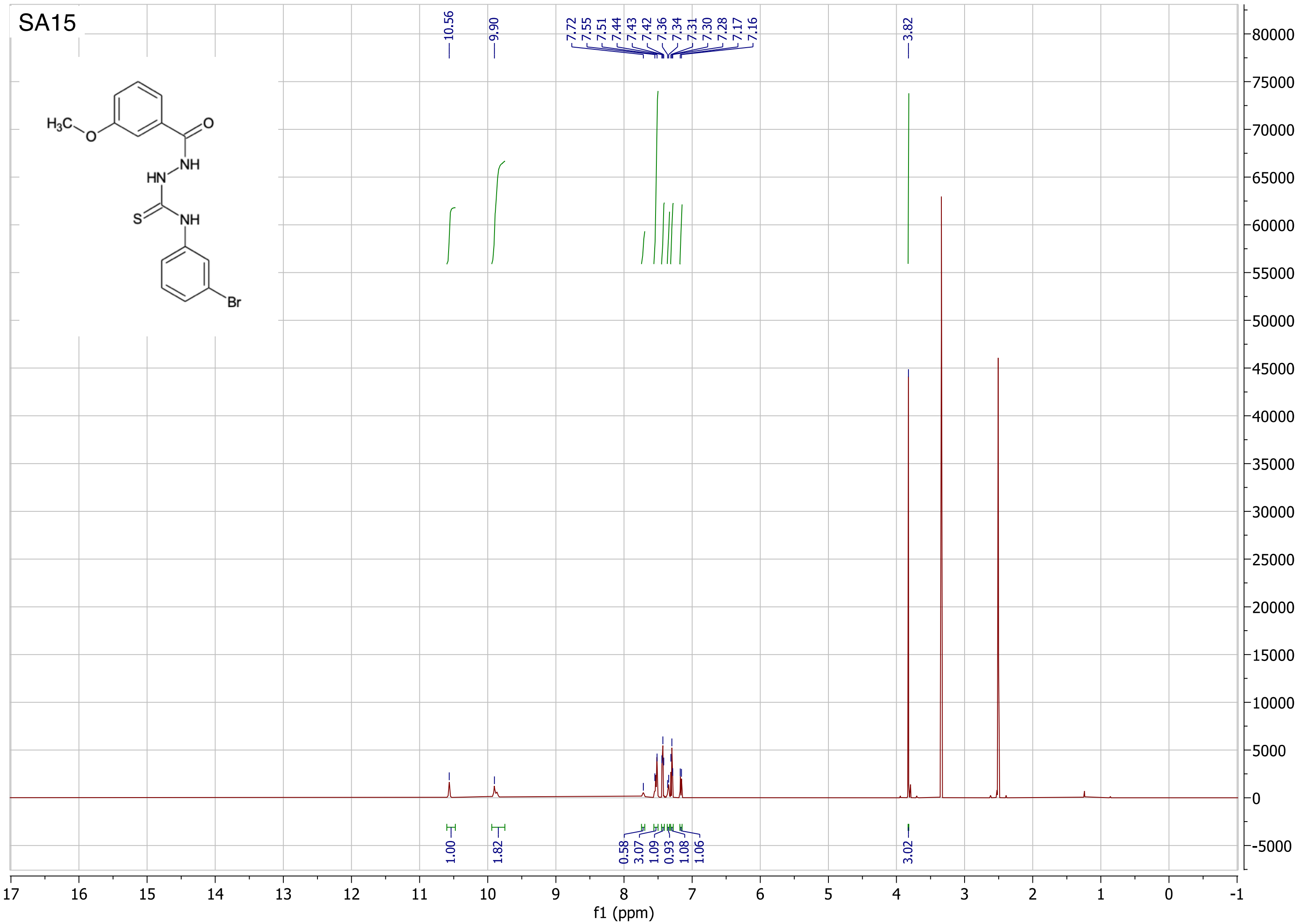

Figure S3. The ^{1}H NMR of compound SA15.

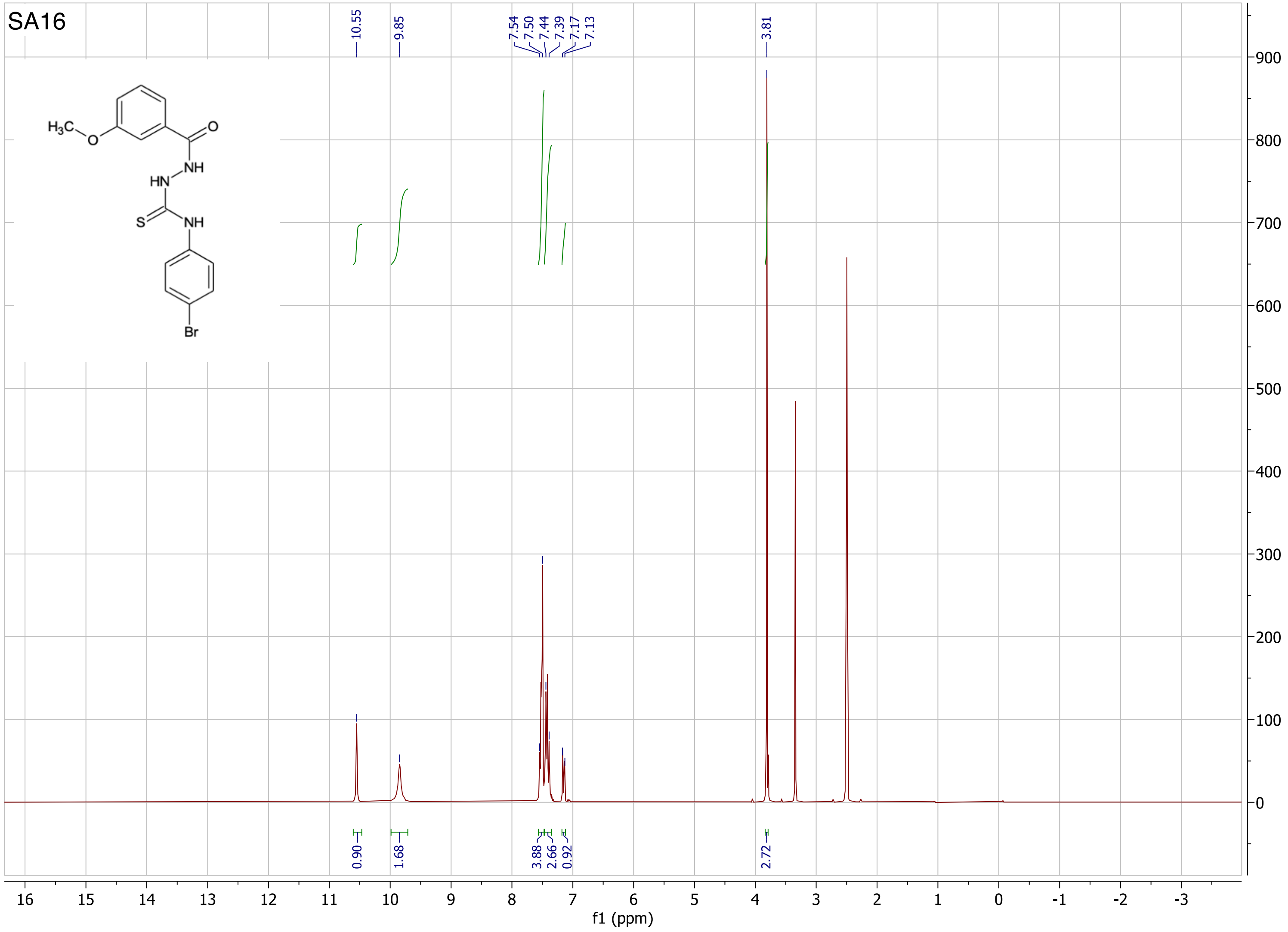

Figure S4. The ^{1}H NMR of compound SA16.

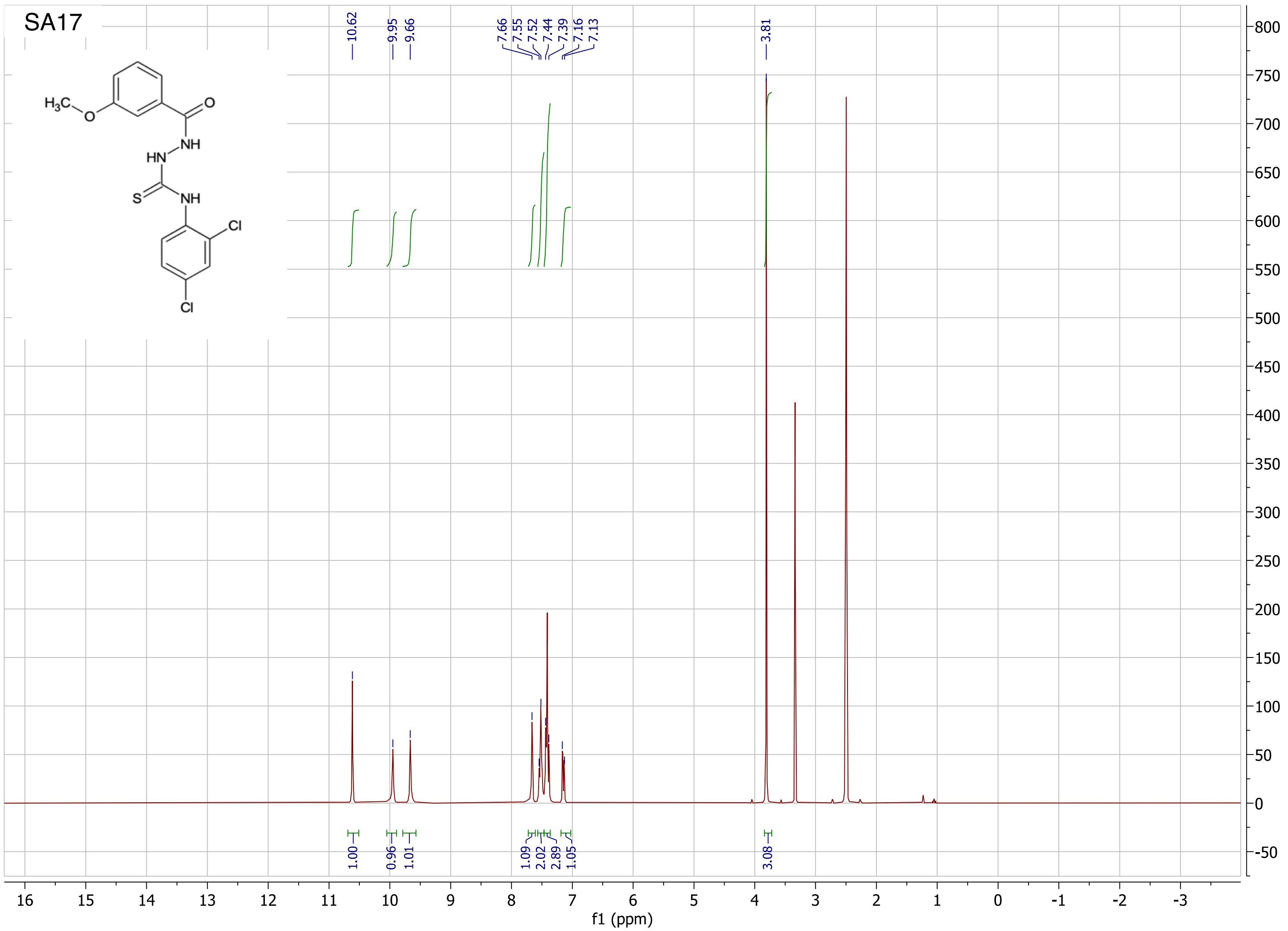

Figure S5. The ^{1}H NMR of compound SA17.

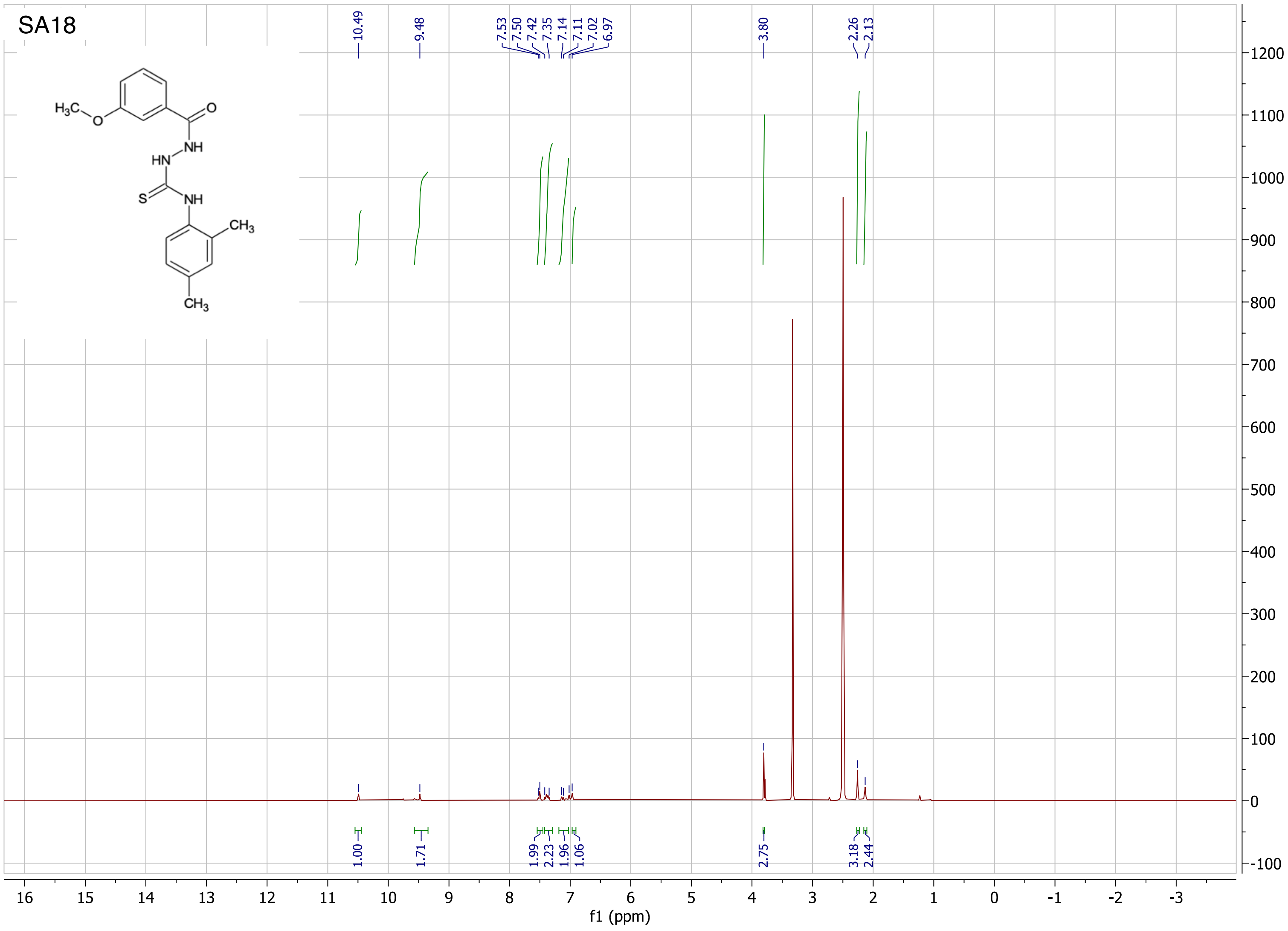

Figure S6. The ¹H NMR of compound SA18.

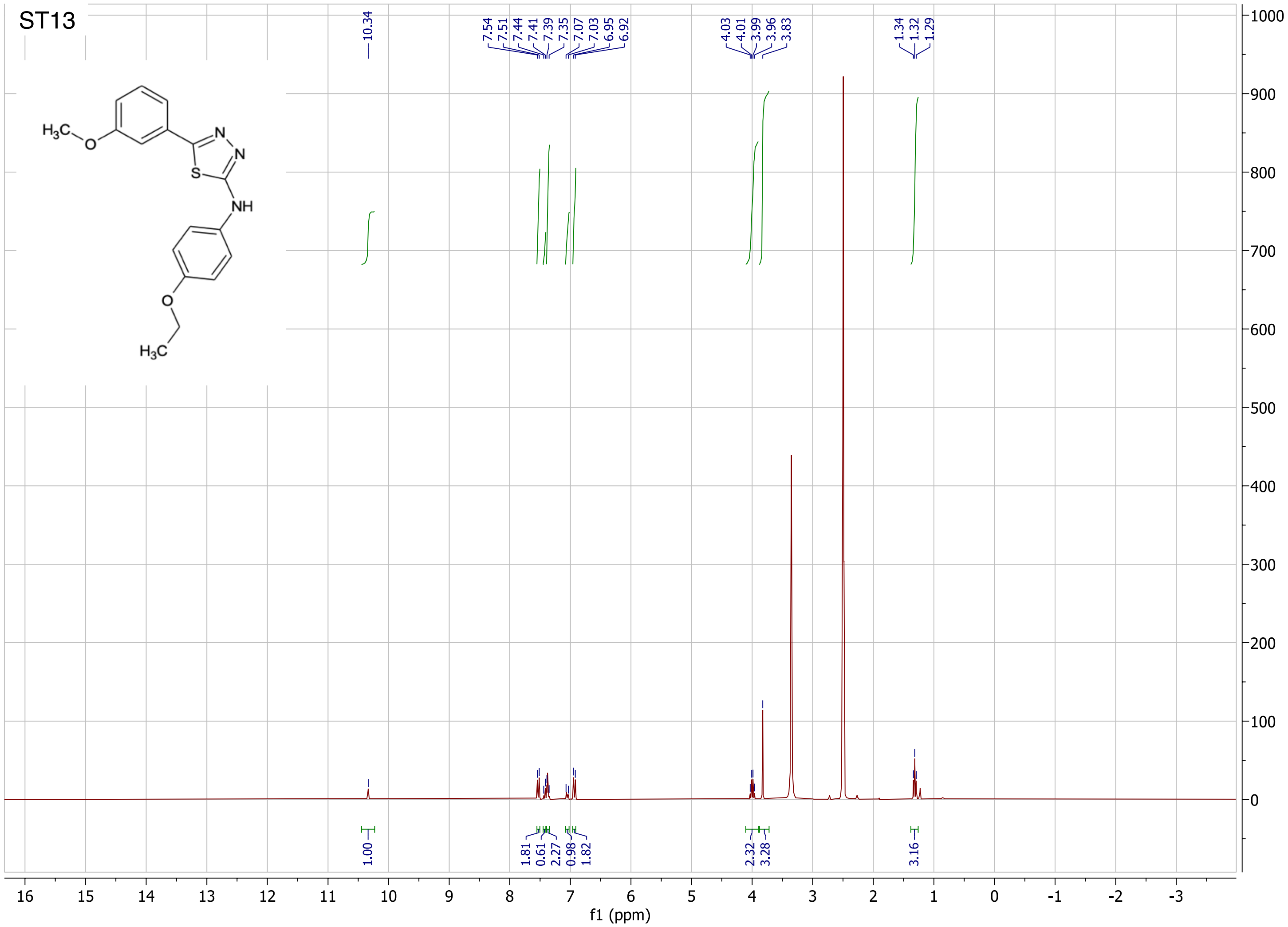

Figure S7. The ^1H NMR of compound ST13.

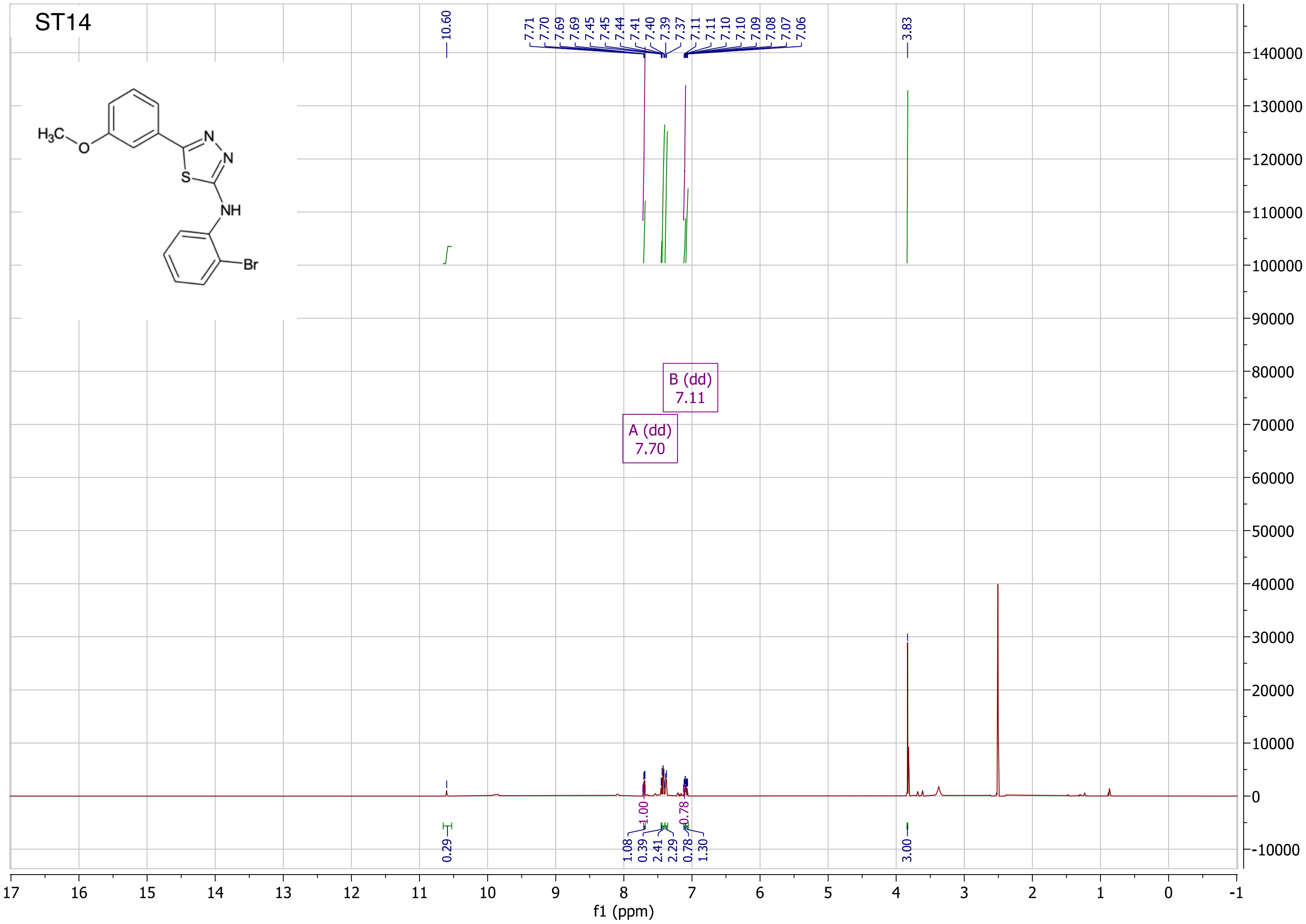

Figure S8. The ^{1}H NMR of compound ST14.

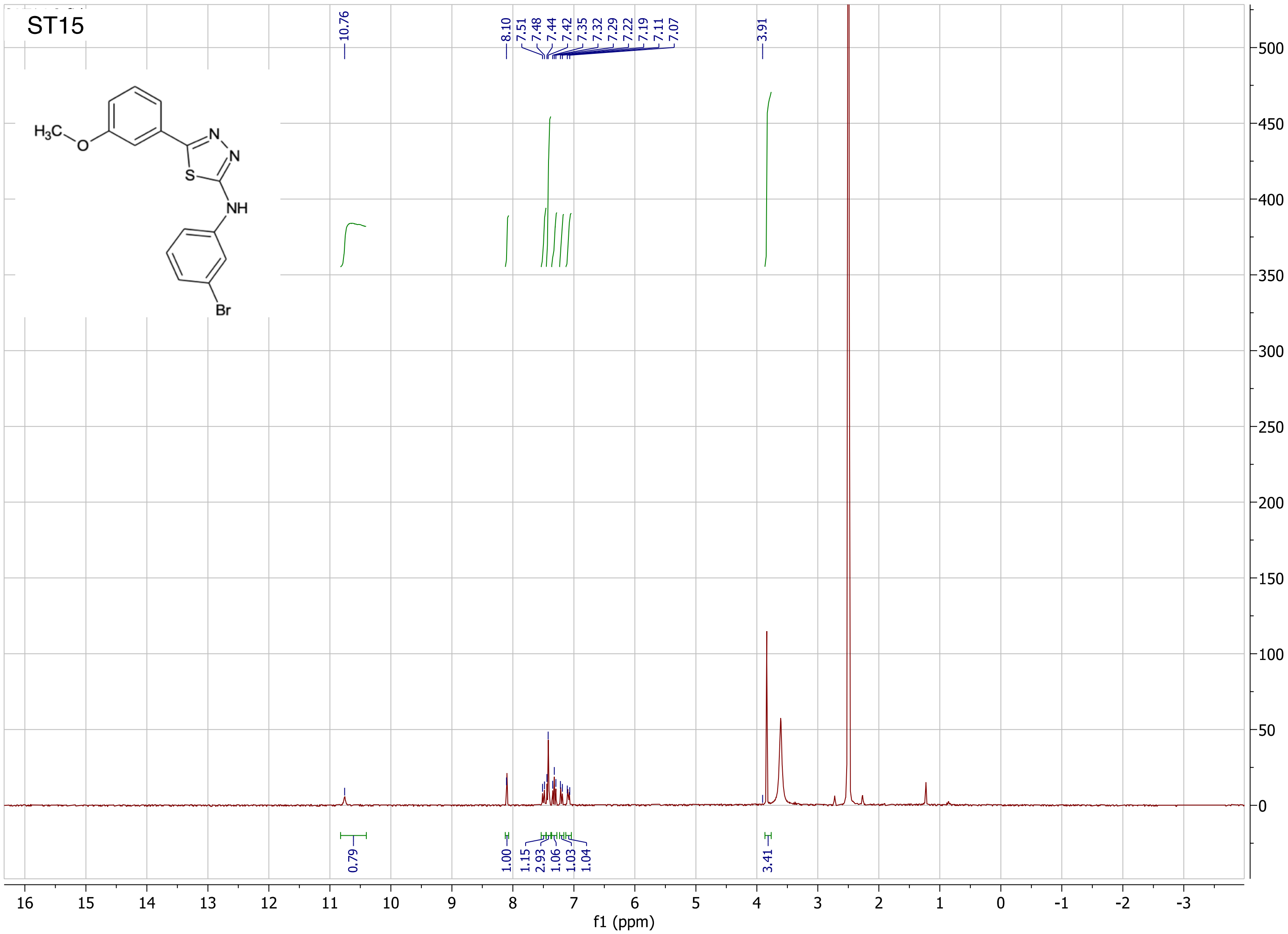

Figure S9. The ^{1}H NMR of compound ST15.

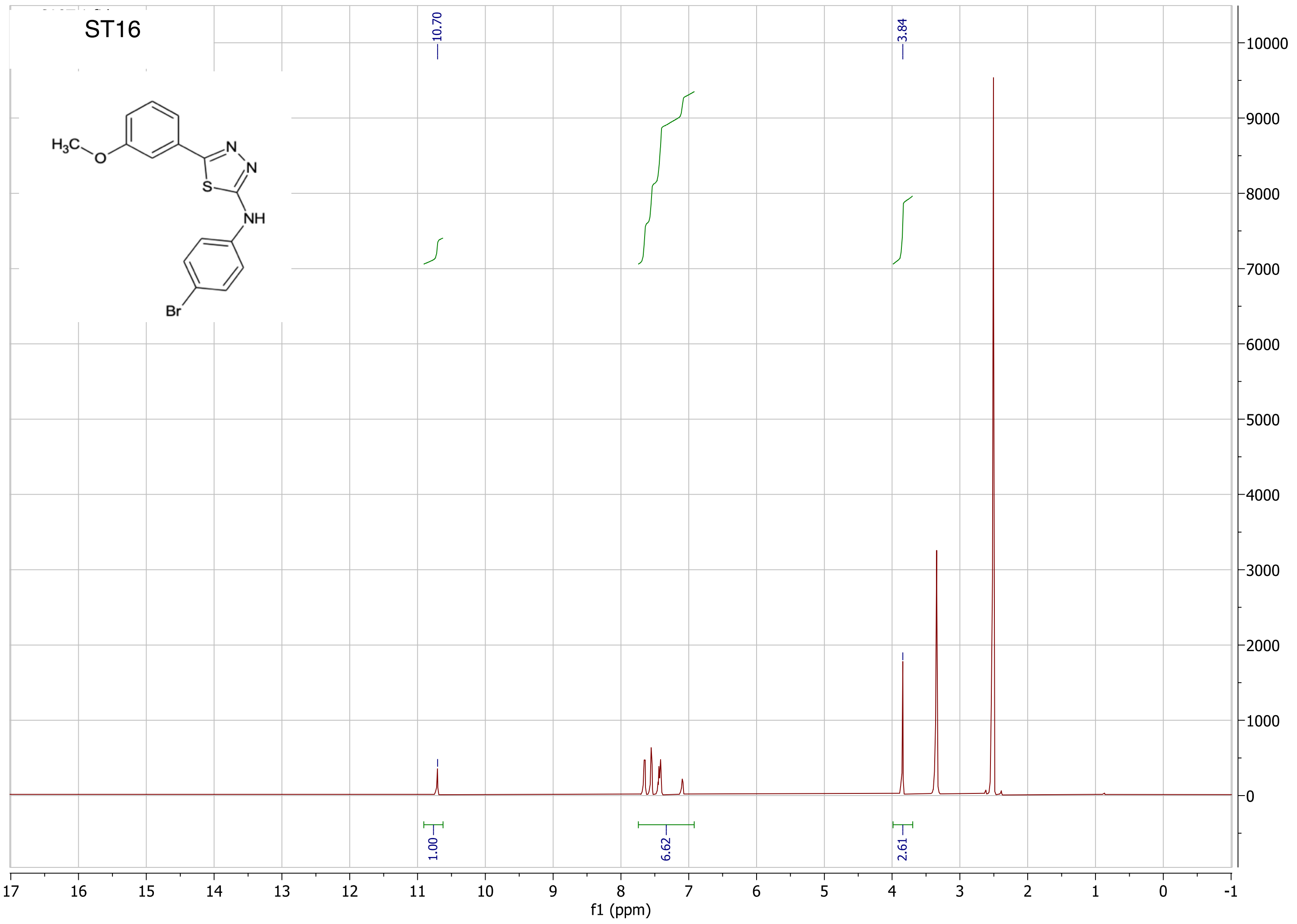

Figure S10. The ^{1}H NMR of compound ST16.

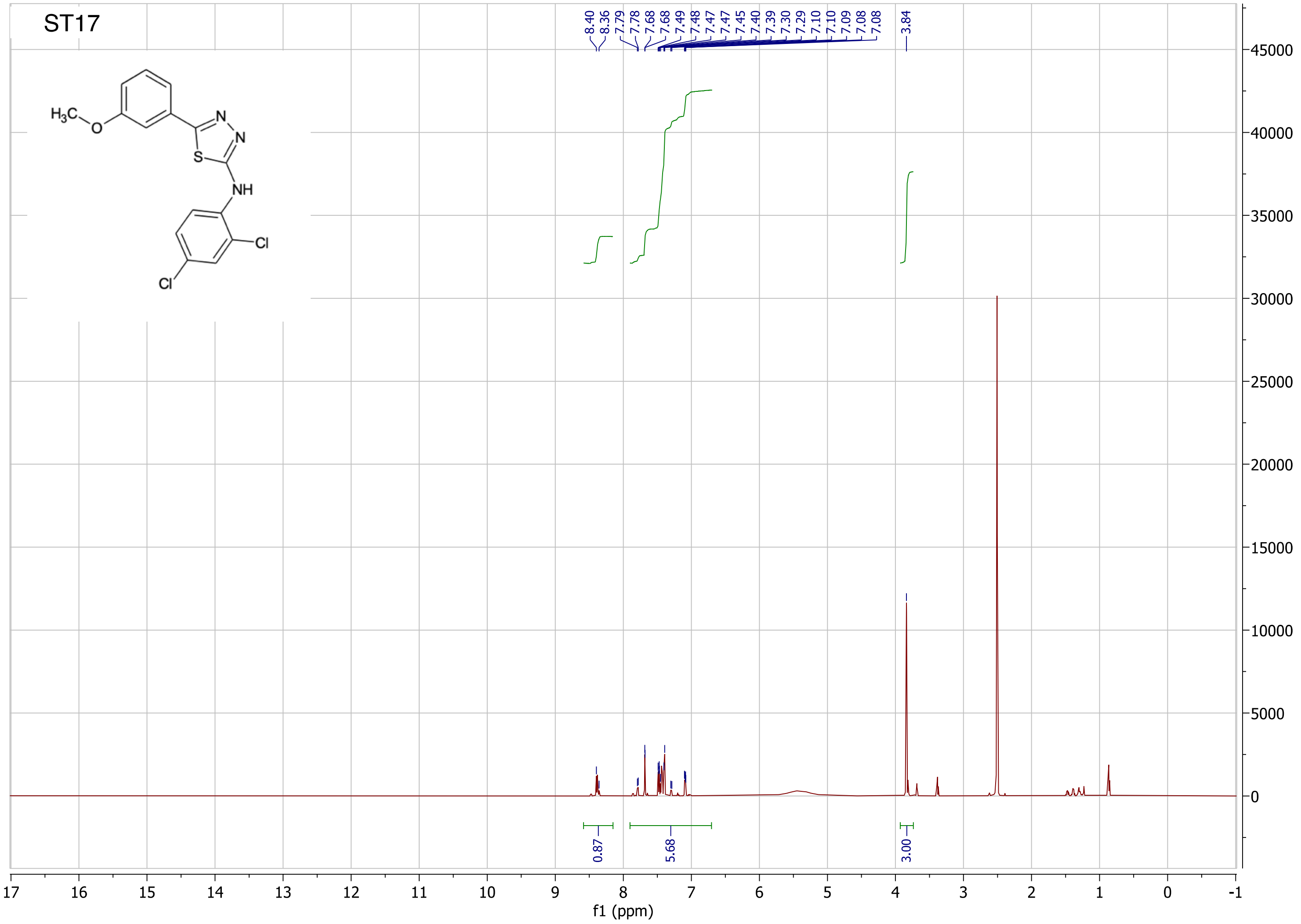

Figure S11. The ¹H NMR of compound ST17.

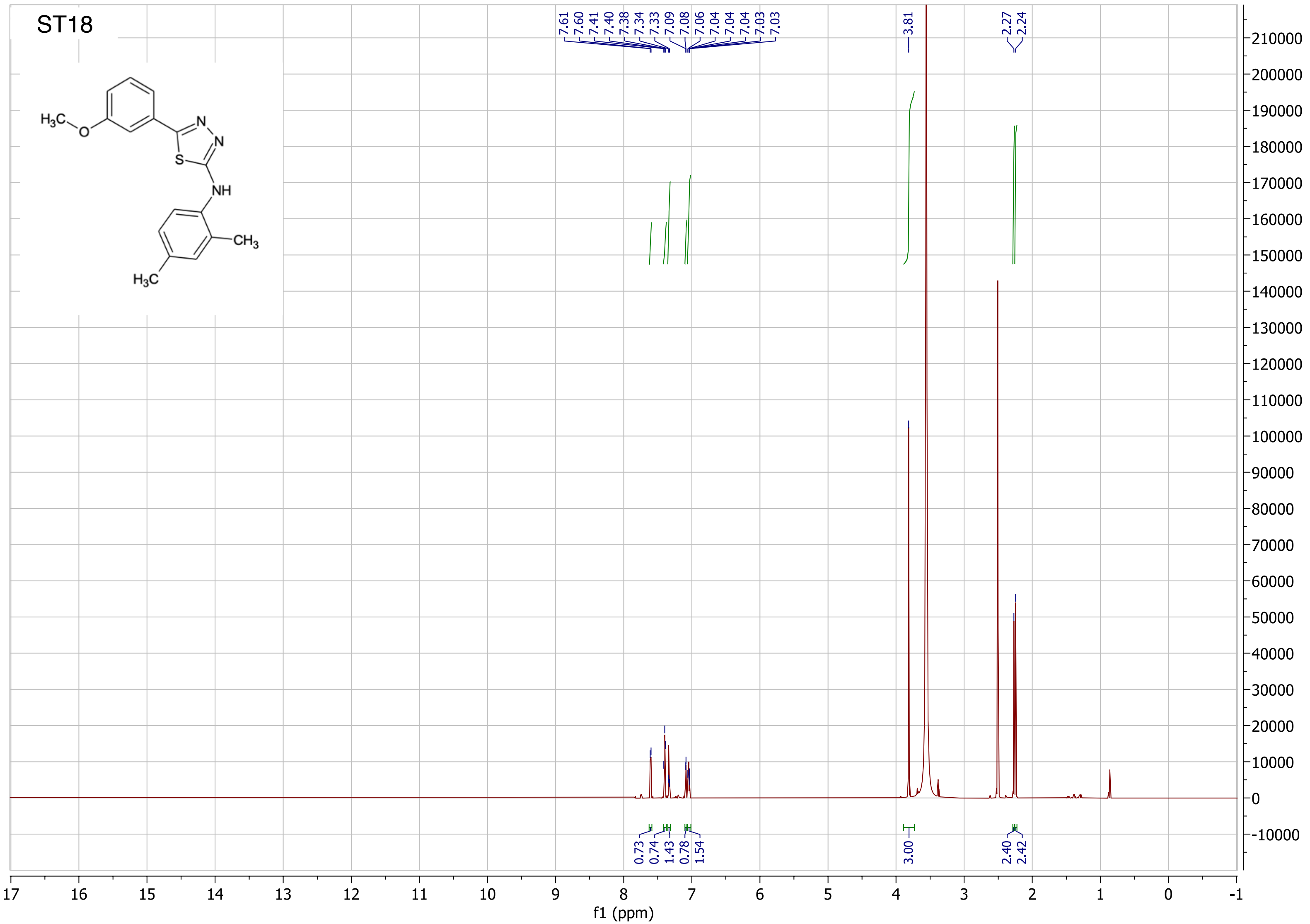

Figure S12. The ^{1}H NMR of compound ST18.

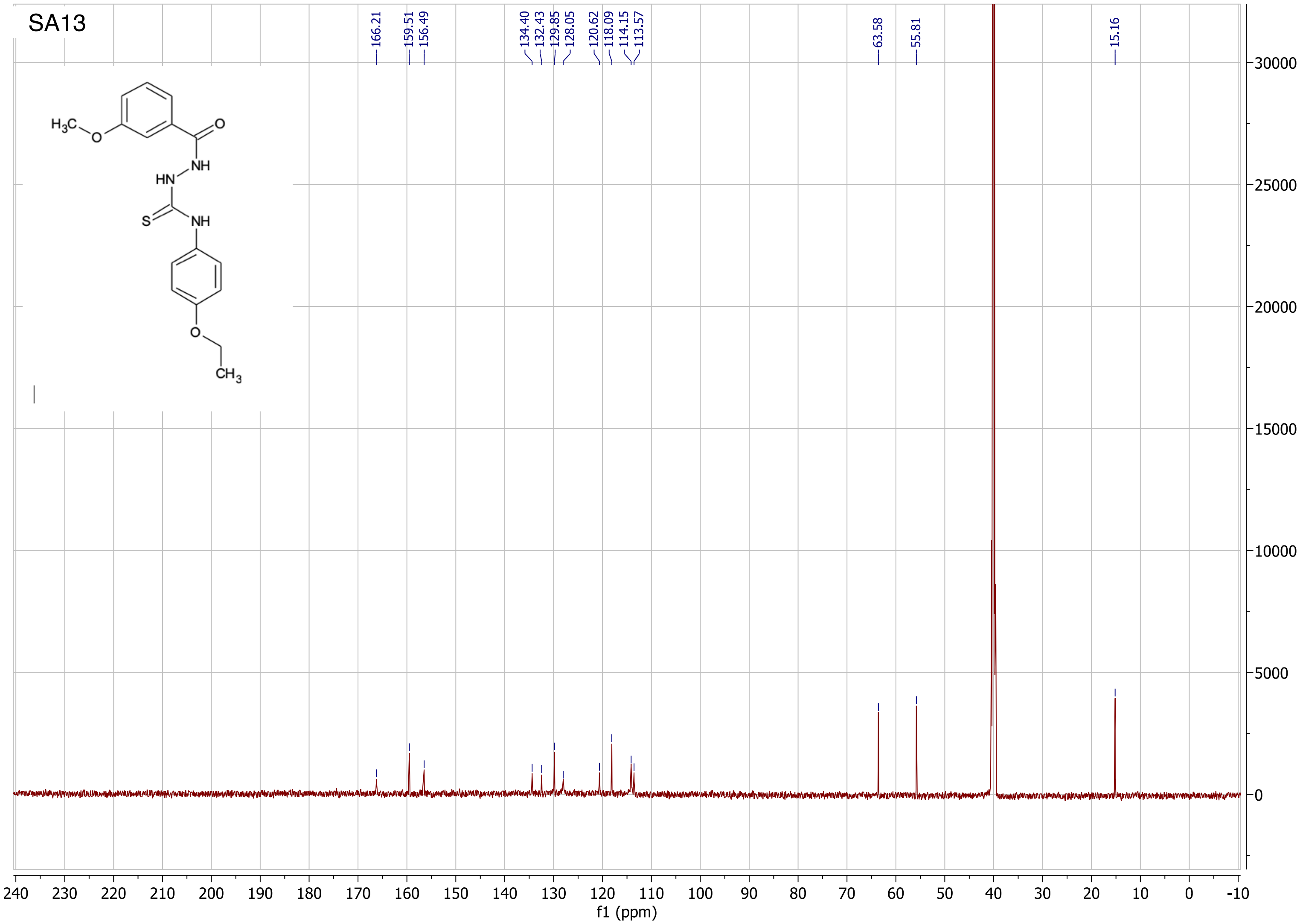

Figure S13. The ^{13}C NMR of compound SA13.

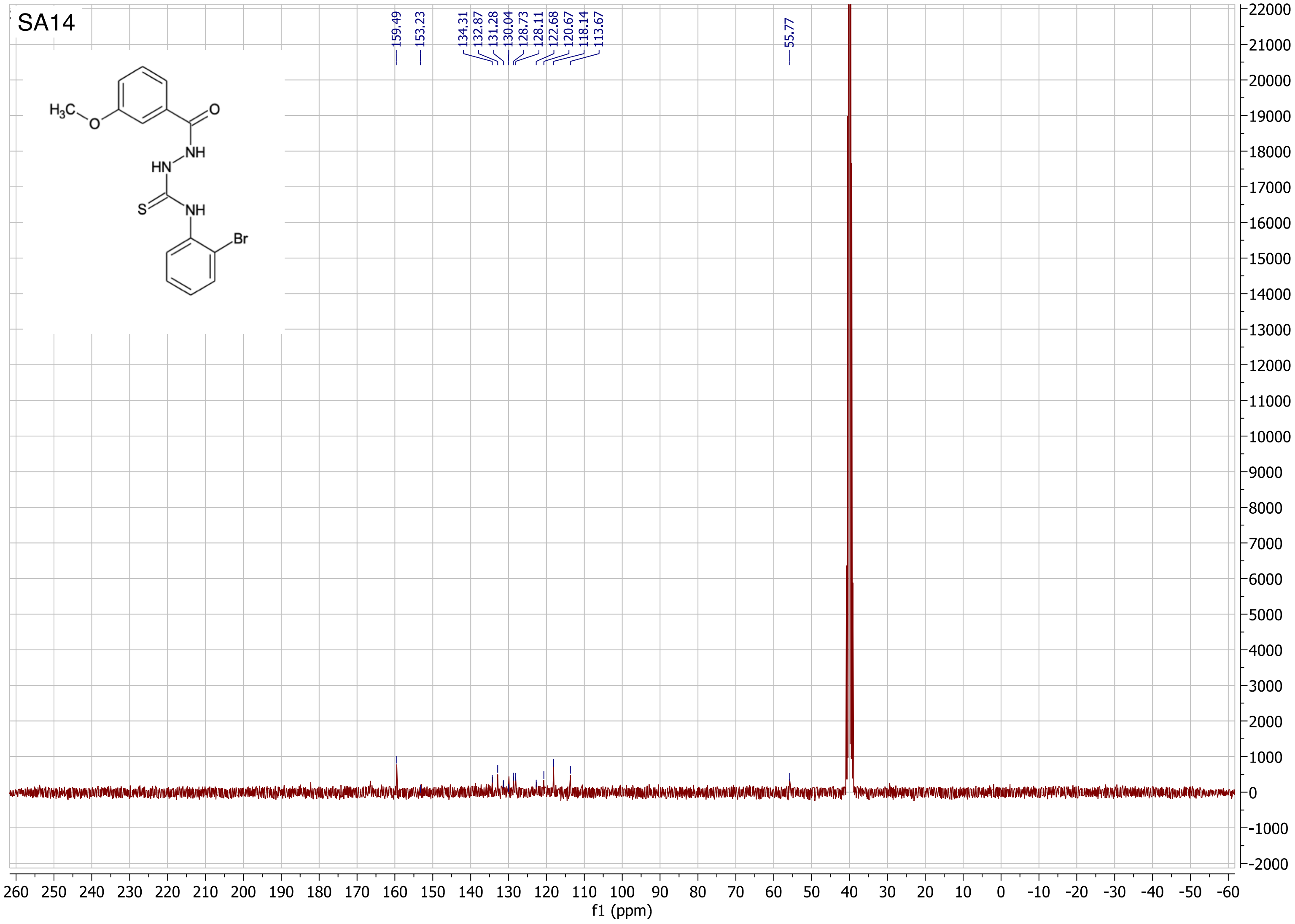

Figure S14. The ^{13}C NMR of compound SA14.

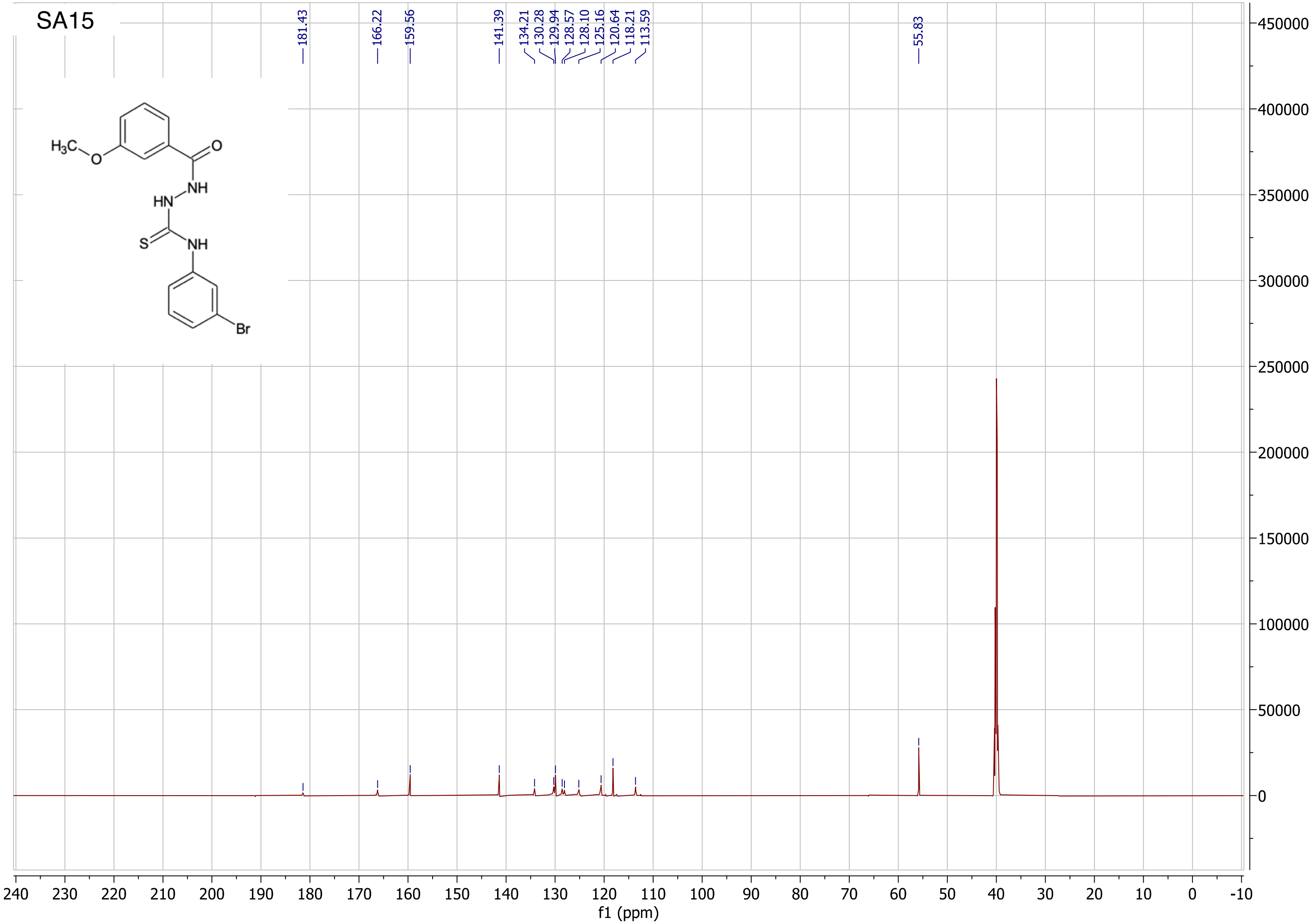

Figure S15. The ^{13}C NMR of compound SA15.

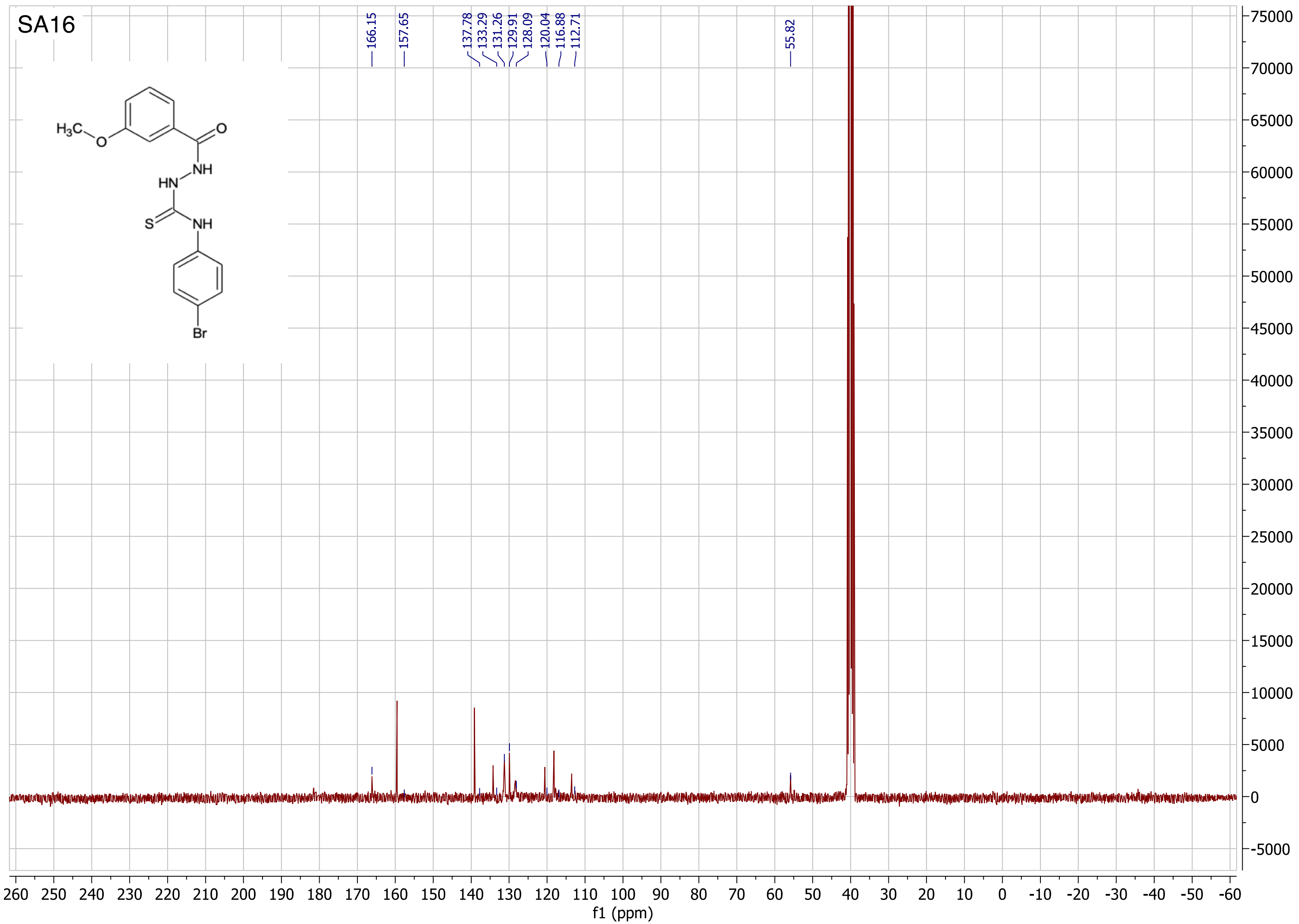

Figure S16. The ^{13}C NMR of compound SA16.

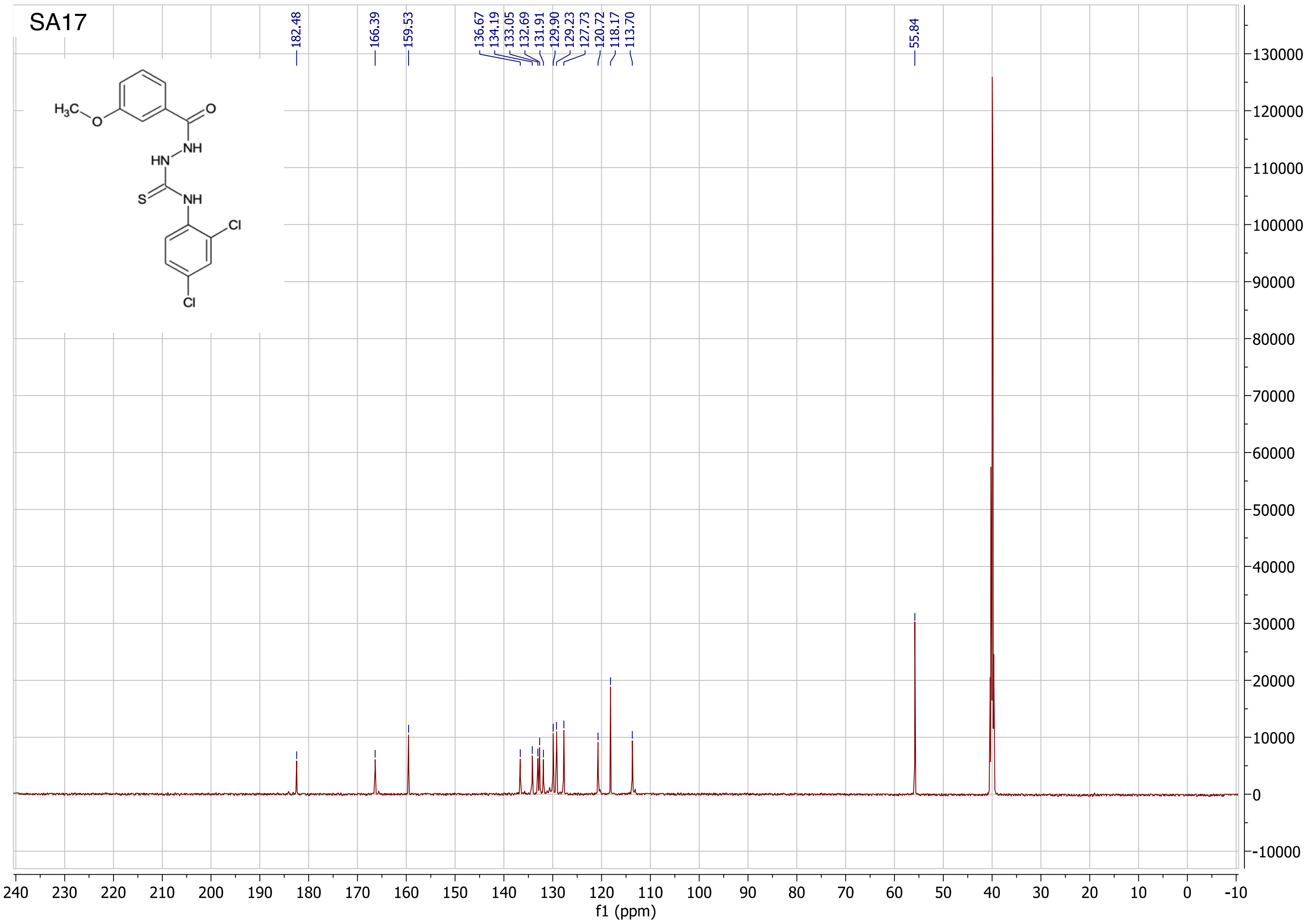

Figure S17. The ^{13}C NMR of compound SA17.

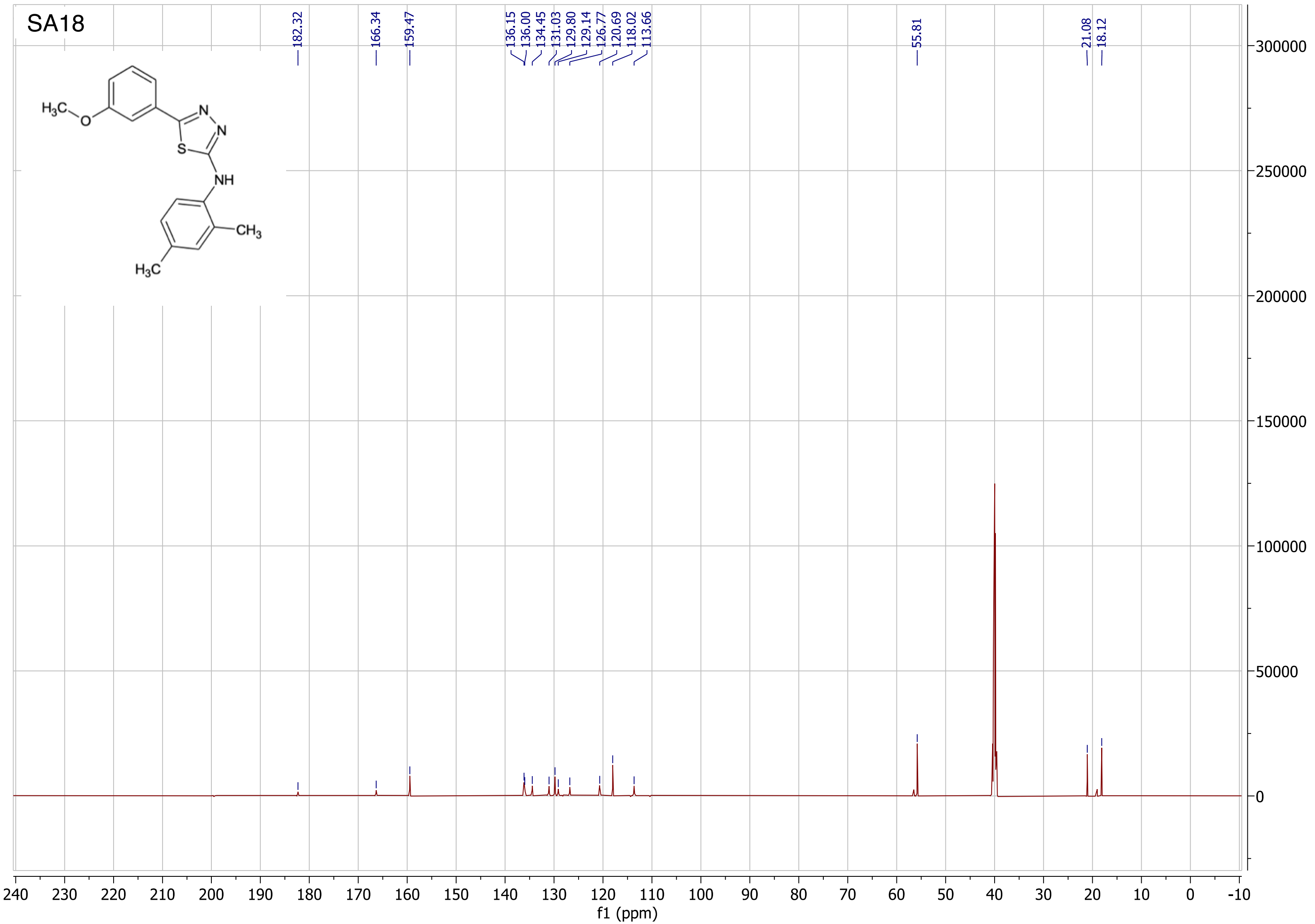

Figure S18. The ^{13}C NMR of compound SA18.

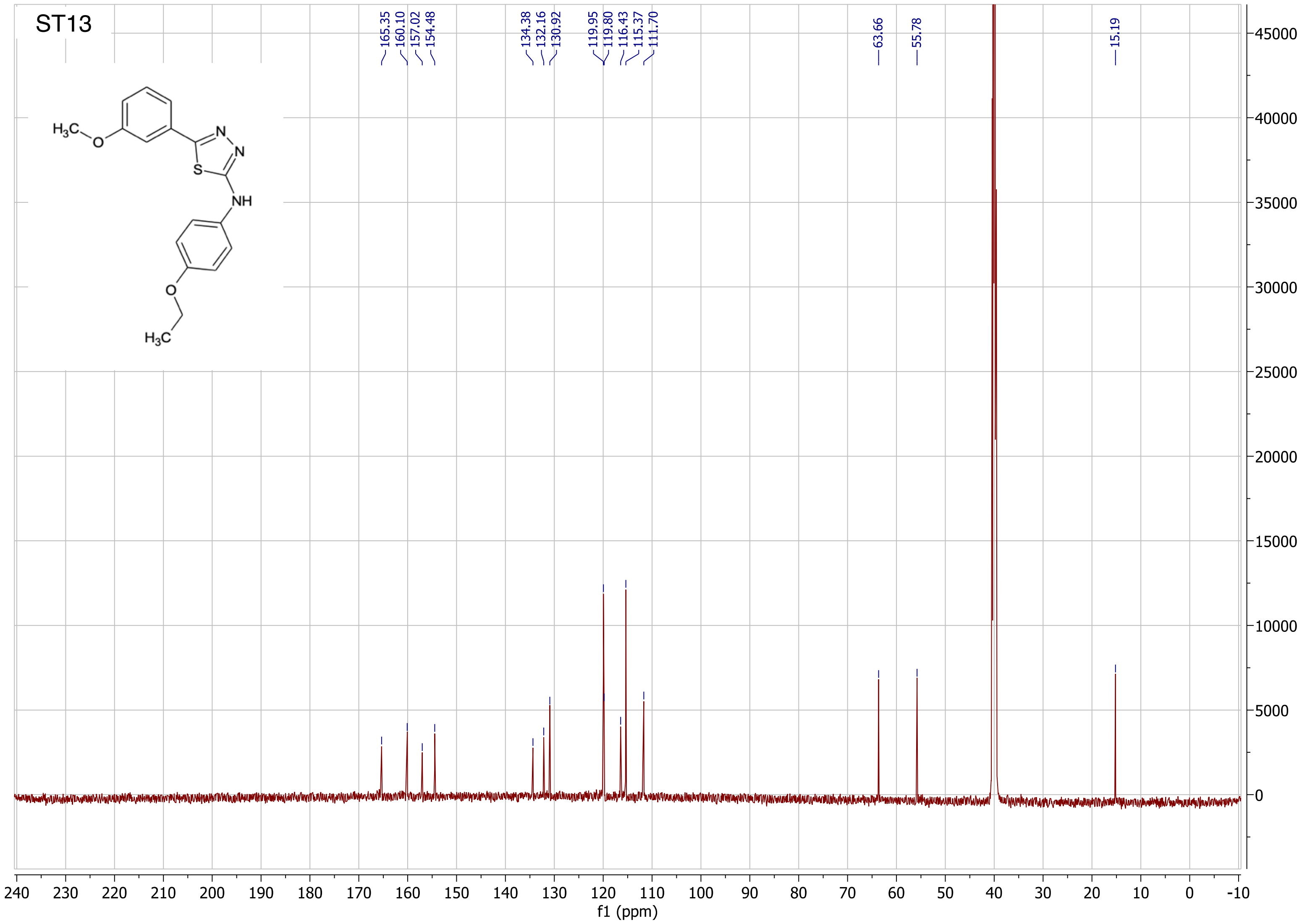

Figure S19. The ^{13}C NMR of compound ST13.

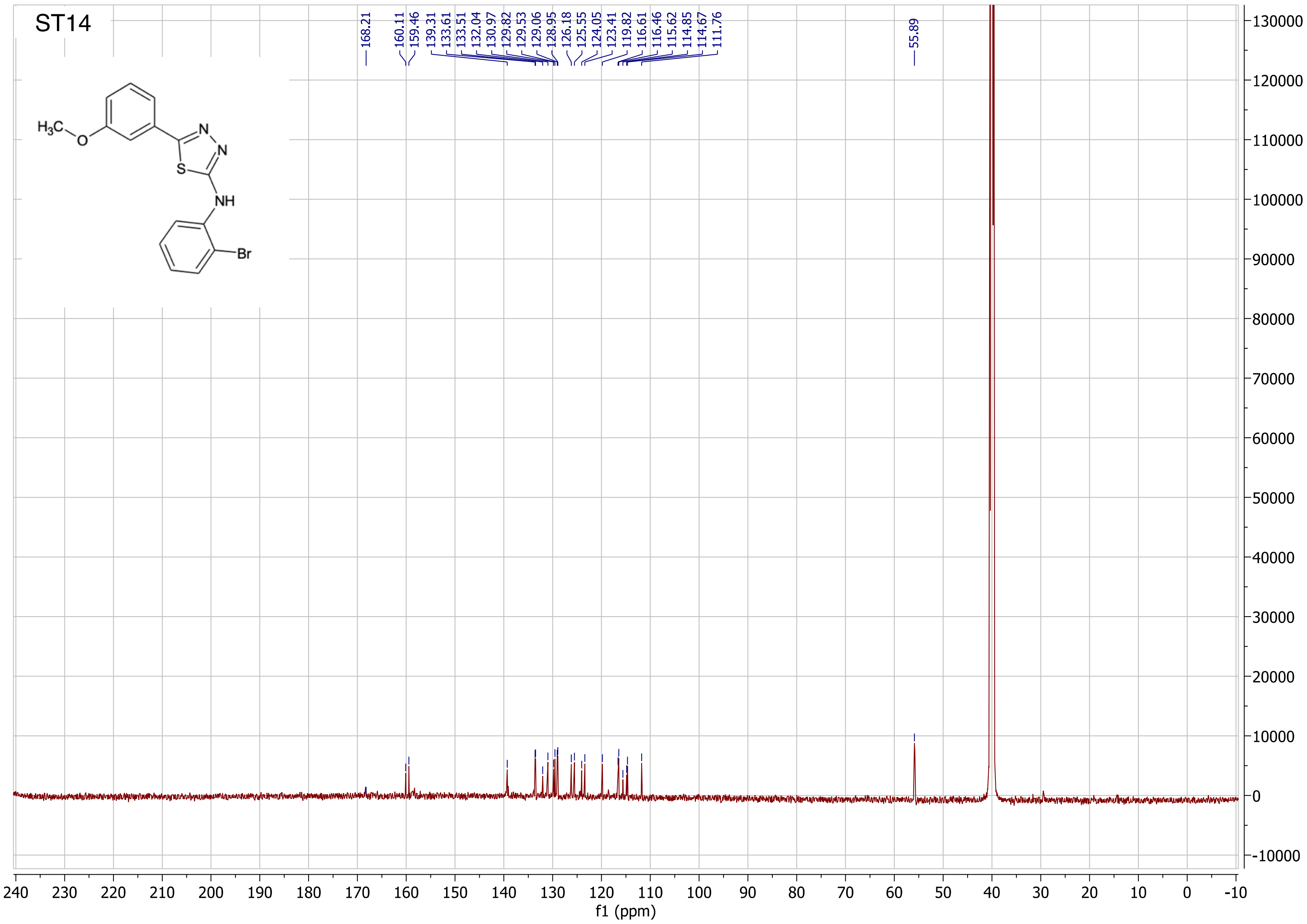

Figure S20. The ^{13}C NMR of compound ST14.

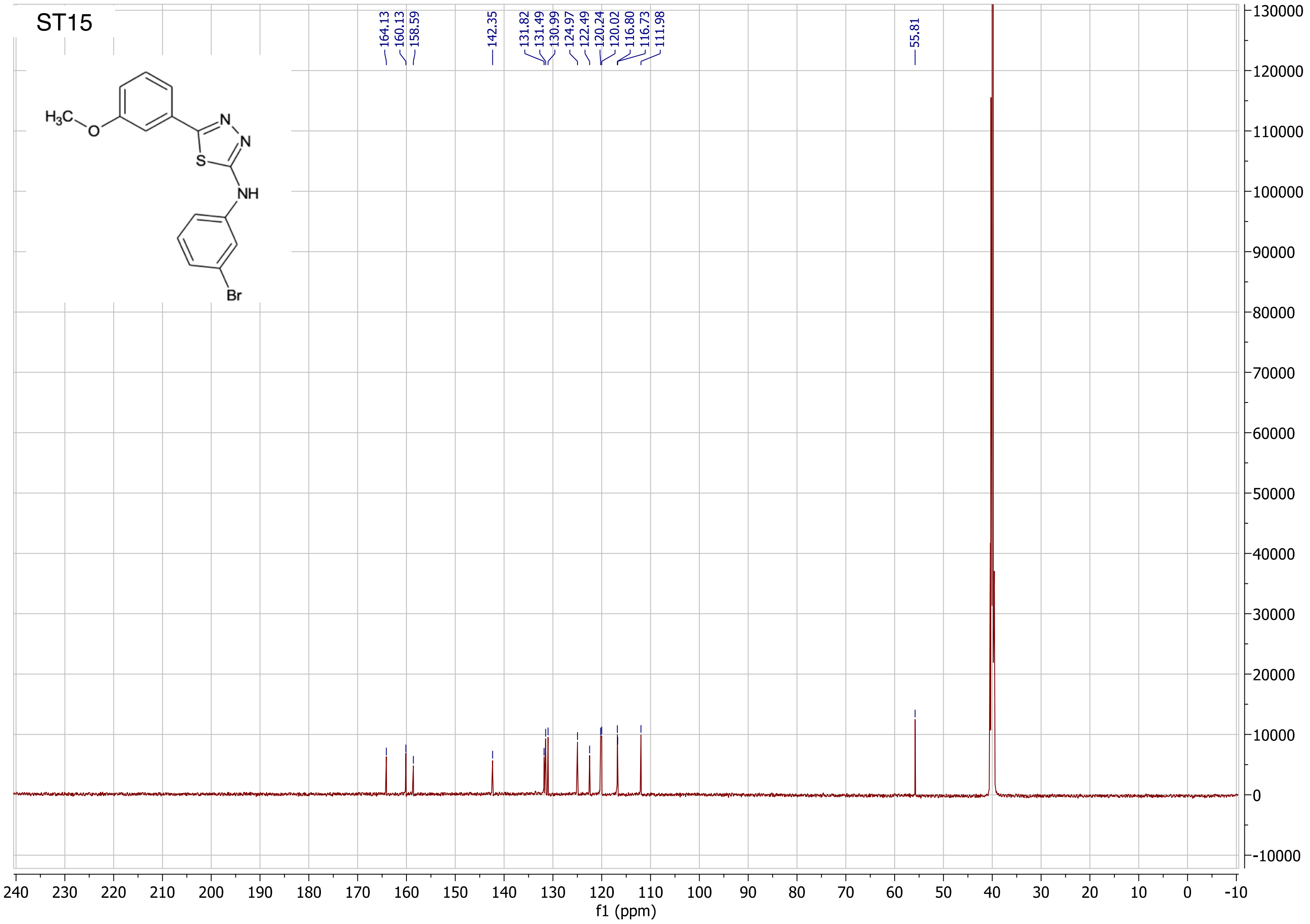

Figure S21. The ^{13}C NMR of compound ST15.

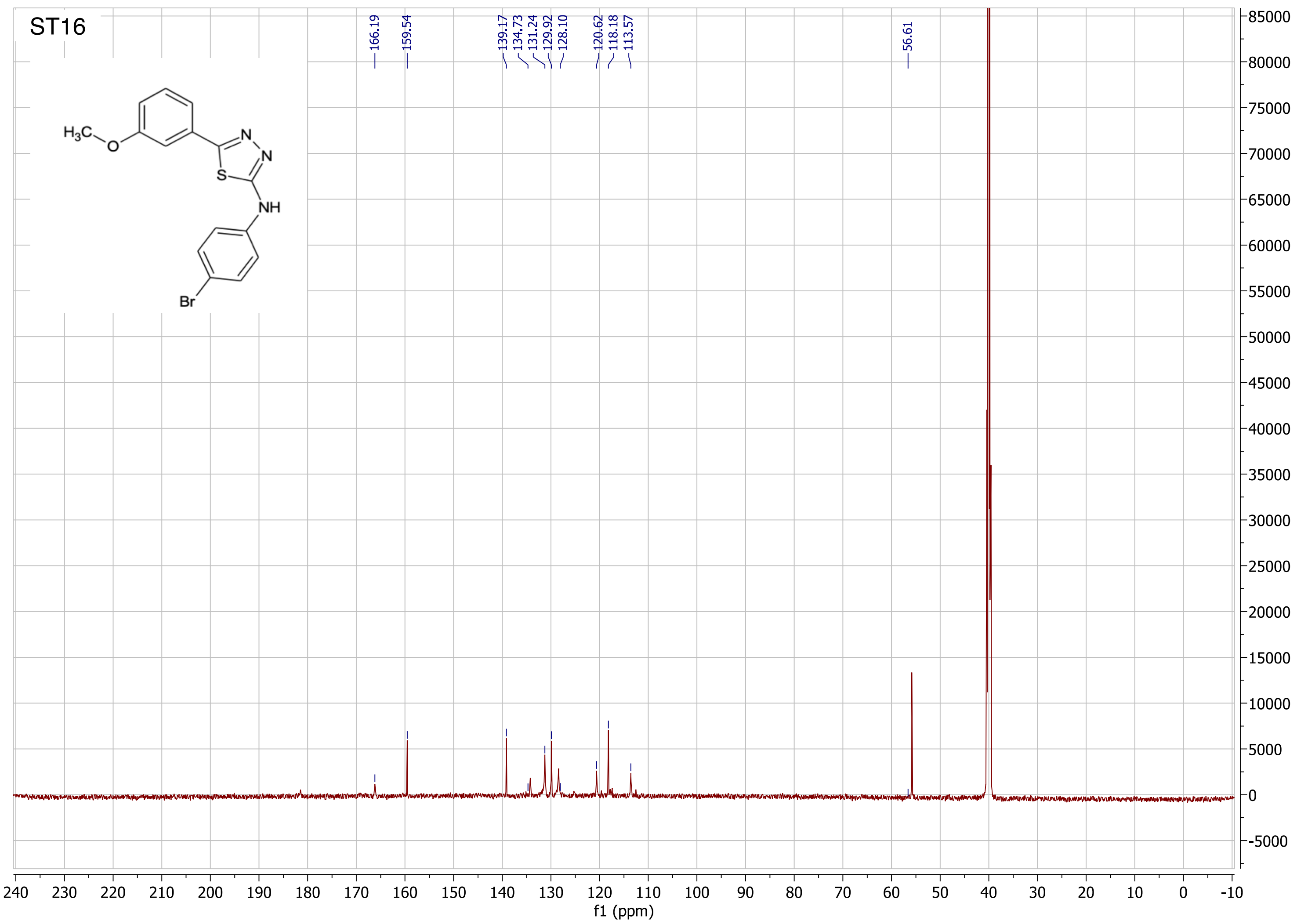

Figure S22. The ^{13}C NMR of compound ST16.

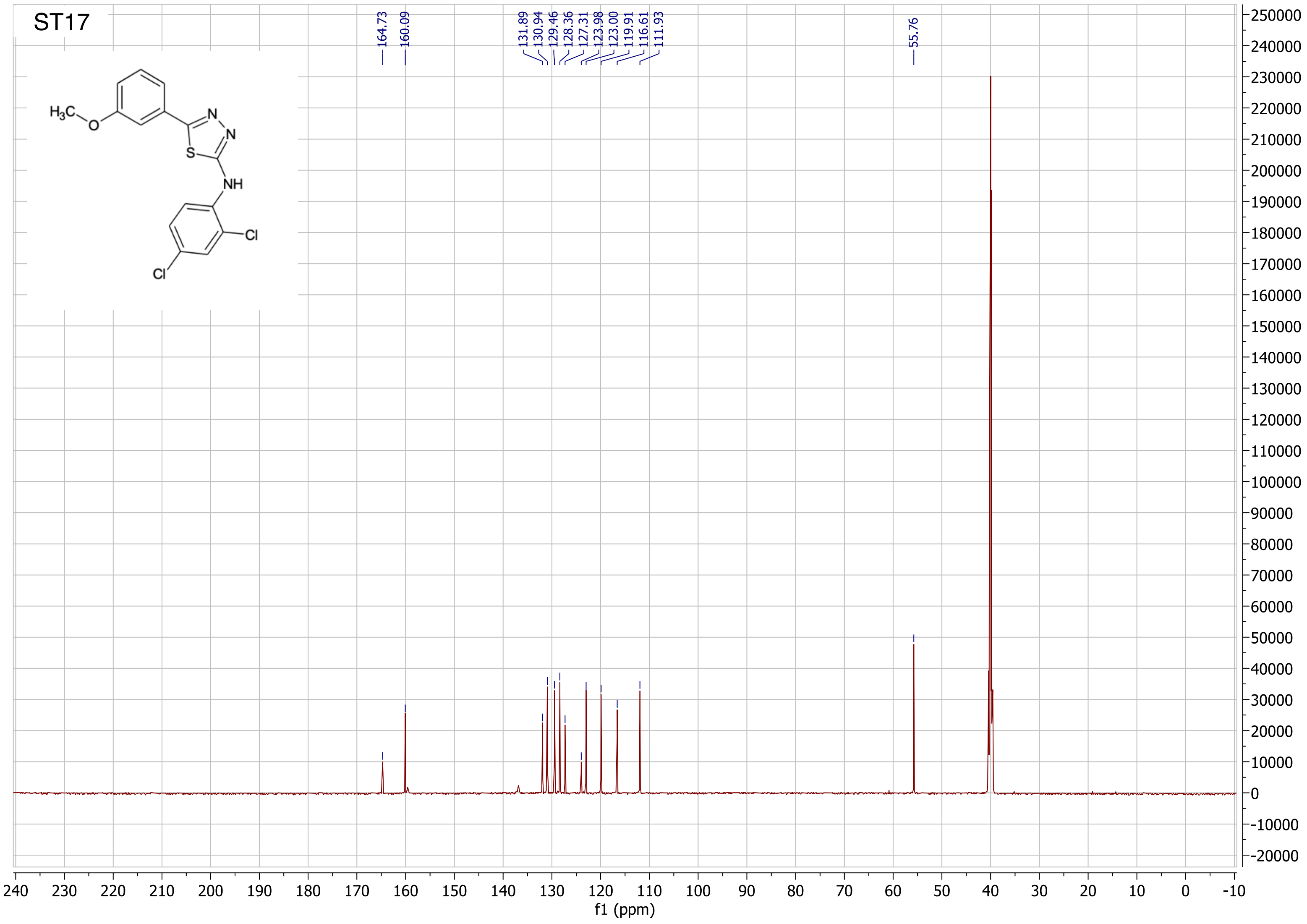

Figure S23. The ^{13}C NMR of compound ST17.

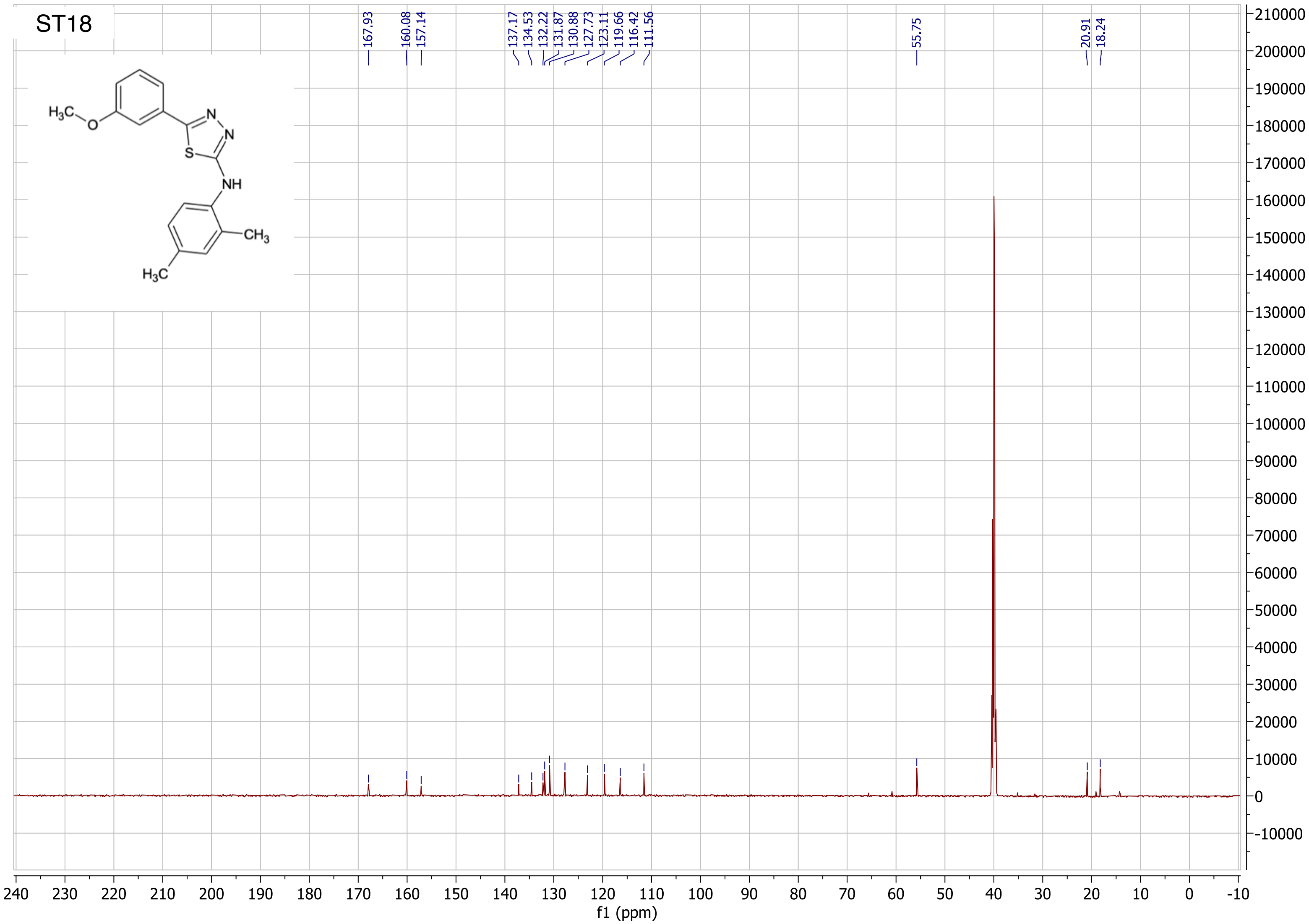

Figure S24. The ^{13}C NMR of compound ST18.

Tabele S1. Antibacterial activity of compounds SA1-18 and ST1-18 against Gram-positive strains.

**Compounds**	*Staphylococcus aureus* **ATCC 25923**	*Staphylococcus aureus* **ATCC 43300**	*Staphylococcus epidermidis* **ATCC 12228**	*Micrococcus luteus* **ATCC 10240**
	**MIC (μg/ml)**			
**SA1**	62.5	62.5	62.5	62.5
**SA2**	1000	>1000	500	62.5
**SA3**	1000	500	125	250
**SA4**	1000	1000	>1000	>1000
**SA5**	125	1000	>1000	>1000
**SA6**	500	1000	250	250
**SA7**	250	1000	62.5	500
**SA10**	250	250	62.5	15.63
**SA11**	250	250	31.25	3.9
**SA12**	500	>1000	125	3.9
**SA 13**	1000	500	125	250
**SA14**	125	250	125	250
**SA15**	500	1000	250	15.63
**SA16**	125	>1000	500	15.63
**SA17**	62.5	>1000	>1000	15.63
**SA18**	>1000	>1000	>1000	>1000

**ST1**	>1000	>1000	>1000	>1000
**ST2**	1000	1000	62.5	500
**ST3**	1000	500	500	250
**ST4**	500	>1000	62.5	31.25
**ST6**	>1000	500	1000	500
**ST7**	>1000	>1000	31.25	31.25
**ST8**	>1000	1000	31.25	500
**ST9**	>1000	1000	1000	1000
**ST10**	1000	1000	1000	1000
**ST11**	>1000	>1000	>1000	>1000
**ST12**	>1000	1000	250	1000
**ST13**	1000	1000	1000	1000
**ST14**	>1000	1000	500	>1000
**ST15**	>1000	500	250	>1000
**ST16**	250	500	31.25	15.63
**ST17**	250	1000	500	250
**ST18**	>1000	>1000	>1000	>1000
**Ciprofloxacin**	0.49	-	0.24	0.98
**Cefuroxime**	-	15.63	0.49	0.98

Table S2. Antibacterial activity of compounds SA1-18 and ST1-18 against Gram-negative strains.

Compounds	*Klebsiella pneumoniae* ATCC 13883	*Escherichia coli* ATCC 25922	*Pseudomonas aeruginosa* ATCC 27853
	**MIC (µg/ml)**		
**SA1**	>1000	>1000	>1000
**SA2**	>1000	>1000	>1000
**SA3**	>1000	1000	>1000
**SA4**	>1000	>1000	>1000
**SA5**	>1000	>1000	>1000
**SA6**	>1000	>1000	>1000
**SA7**	>1000	>1000	>1000
**SA10**	>1000	>1000	>1000
**SA11**	>1000	>1000	>1000
**SA12**	>1000	>1000	>1000
**SA 13**	>1000	>1000	>1000
**SA14**	>1000	>1000	>1000
**SA15**	>1000	>1000	>1000
**SA16**	>1000	>1000	>1000
**SA17**	>1000	>1000	>1000
**SA18**	>1000	>1000	>1000
**ST1**	>1000	>1000	>1000

**ST2**	>1000	>1000	>1000
**ST3**	1000	1000	1000
**ST4**	>1000	>1000	>1000
**ST6**	>1000	>1000	>1000
**ST7**	>1000	>1000	>1000
**ST8**	>1000	>1000	>1000
**ST9**	>1000	>1000	>1000
**ST10**	>1000	>1000	>1000
**ST11**	>1000	>1000	>1000
**ST12**	>1000	>1000	>1000
**ST13**	>1000	>1000	>1000
**ST14**	>1000	>1000	>1000
**ST15**	>1000	>1000	>1000
**ST16**	>1000	>1000	>1000
**ST17**	>1000	>1000	>1000
**ST18**	>1000	>1000	>1000
**Ciprofloxacin**	0.36	0.024	0.72
